# Bone Marrow-Derived Mesenchymal Stem Cell-Laden Nanocomposite Scaffolds Enhance Bone Regeneration in Rabbit Critical-Size Segmental Bone Defect Model

**DOI:** 10.3390/jfb15030066

**Published:** 2024-03-10

**Authors:** Elangovan Kalaiselvan, Swapan Kumar Maiti, Shivaraju Shivaramu, Shajahan Amitha Banu, Khan Sharun, Divya Mohan, Sangeetha Palakkara, Sadhan Bag, Monalisa Sahoo, Suresh Ramalingam, Jürgen Hescheler

**Affiliations:** 1 Division of Surgery, ICAR-Indian Veterinary Research Institute, Izatnagar, Bareilly 243122, India; selvanholmes@gmail.com (E.K.); shivaraju558@gmail.com (S.S.); aamianchu@gmail.com (S.A.B.); sharunkhansk@gmail.com (K.S.); divyadhruvam@gmail.com (D.M.); sangeethapalakkara@gmail.com (S.P.); 2 Department of Veterinary Surgery and Radiology, Tamil Nadu Veterinary and Animal Sciences University, Chennai 600007, India; 3 Graduate Institute of Medicine, Yuan Ze University, Taoyuan 32003, Taiwan; 4 Eastern Regional Station, ICAR-Indian Veterinary Research Institute, Kolkata 700037, India; bag658@gmail.com; 5 Division of Pathology, ICAR-Indian Veterinary Research Institute, Izatnagar, Bareilly 243122, India; vety.lisa@gmail.com; 6 Department of Animal Nutrition, Tamil Nadu Veterinary and Animal Sciences University, Chennai 600007, India; sureshbpmynepolean@gmail.com; 7 Institute of Neurophysiology, University of Cologne, 50931 Cologne, Germany; j.hescheler@uni-koeln.de

**Keywords:** bone engineering, hydroxyapatite, nanomaterial scaffolds, nanoscaffolding, regenerative medicine, tissue engineering

## Abstract

Bone regeneration poses a significant challenge in the field of tissue engineering, prompting ongoing research to explore innovative strategies for effective bone healing. The integration of stem cells and nanomaterial scaffolds has emerged as a promising approach, offering the potential to enhance regenerative outcomes. This study focuses on the application of a stem cell-laden nanomaterial scaffold designed for bone regeneration in rabbits. The in vivo study was conducted on thirty-six healthy skeletally mature New Zealand white rabbits that were randomly allocated into six groups. Group A was considered the control, wherein a 15 mm critical-sized defect was created and left as such without any treatment. In group B, this defect was filled with a polycaprolactone–hydroxyapatite (PCL + HAP) scaffold, whereas in group C, a PCL + HAP-carboxylated multiwalled carbon nanotube (PCL + HAP + MWCNT-COOH) scaffold was used. In group D, a PCL + HAP + MWCNT-COOH scaffold was used with local injection of bone morphogenetic protein-2 (BMP-2) on postoperative days 30, 45, and 60. The rabbit bone marrow-derived mesenchymal stem cells (rBMSCs) were seeded onto the PCL + HAP + MWCNT-COOH scaffold by the centrifugal method. In group E, an rBMSC-seeded PCL + HAP + MWCNT-COOH scaffold was used along with the local injection of rBMSC on postoperative days 7, 14, and 21. For group F, in addition to the treatment given to group E, BMP-2 was administered locally on postoperative days 30, 45, and 60. Gross observations, radiological observation, scanning electron microscopic assessment, and histological evaluation study showed that group F displayed the best healing properties, followed by group E, group D, group C, and B. Group A showed no healing with ends blunting minimal fibrous tissue. Incorporating growth factor BMP-2 in tissue-engineered rBMSC-loaded nanocomposite PCL + HAP + MWCNT-COOH construct can augment the osteoinductive and osteoconductive properties, thereby enhancing the healing in a critical-sized bone defect. This novel stem cell composite could prove worthy in the treatment of non-union and delayed union fractures in the near future.

## 1. Introduction

Bone loss can result from various factors such as trauma, degeneration, and the surgical removal of bone-borne tumors. Often, the size of larger defects surpasses the healing capacity of the body’s natural healing mechanism [1]. These intraosseous wounds that do not heal spontaneously are referred to as critical-size defects [2]. Therefore, to replace the bone that ceases to regenerate spontaneously, the substitute must possess osteoinductive, osteogenic, and osteoconductive properties [3]. Strategies to address non-union in critical-size defects include the use of implants, bone grafting, or cellular therapy alone or in combination [4]. However, implants used alone can have deleterious effects such as stress shielding and osteopenia. Autografting, a long-standing gold standard method, has limitations such as a limited donor site, associated morbidity, and the need for multiple surgeries [5]. Allogeneic grafting poses challenges related to immunogenicity and disease transmission [6].

Biomaterials have been employed to mitigate these drawbacks, along with advancements in bioengineering technology. Acting as a scaffold for a temporary extracellular matrix, biomaterials support cell viability and can release or stimulate signaling molecules at the desired site. However, providing an exact healing microenvironment akin to the normal physiological process remains challenging [7]. Bioactive ceramics used as a scaffold in bone defects include tricalcium phosphate (TCP), aluminum–calcium–phosphorus oxide (ALCAP), calcium hydroxyapatite (HA), zinc–calcium–phosphorus oxide (ZCAP), etc. The most obvious drawback in the orthopedic use of ceramics as scaffolding and fixation material is their inherent brittleness [8]. Biopolymers offer several advantages over bioglass and bioceramics, including enhanced stability, ease of manufacturing, and the option for both synthetic and natural sources. These attributes make biopolymers more versatile and adaptable for various biomedical applications. The polymers are divided into natural and synthetic. For natural polymers, the polymers are starch, alginate, collagen, fibrin, and silk. Synthetic polymers such as polylactide-co-glycolide (PLGA) and polycaprolactone (PCL) are most commonly used in biomaterial bone regeneration studies [9]. However, these polymers alone have demonstrated limited osteoconductive or osteoinductive properties [10]. Hence, the integration of biological processing with bioengineering techniques becomes essential to develop biomaterials that mimic natural bone properties and facilitate cell implantation, thereby supporting the healing process.

There are various methods in bioengineering technology to fabricate the scaffold, namely solvent casting, gas foaming, emulsification, freeze-drying, phase separation, electrospinning, and 3D bioprinting. Among the above stated, electrospinning is simple and develops a scaffold with a large surface area [11]. Electrospinning is a versatile technique commonly used in bio-tissue engineering to produce nanoscale materials [12]. It produces ultrafine, high-surface-area, non-oven polymeric fibers [13]. The morphology of electrospun mats closely mimics the structure of the extracellular matrix (ECM) found in the body [14]. The blending of multiple polymers can make it possible to develop composite scaffolds using electrospinning technique [15]. An advantage of electrospun scaffolds is their ability to bind, release, and activate signaling molecules [16]. Nanocomposite MWCNT (multiwalled carbon nanotube)-PCL (polycaprolactone) scaffolds are found to be ideal for bioengineering applications [17].

Despite its widespread use in regenerative medicine, PCL has limited ability to induce adhesion, proliferation, and osteogenic cell differentiation [18]. This constraint can be overcome by incorporating different calcium phosphates, such as HA or TCP [18,19,20]. Previous in vitro studies have assessed the potential of 3D-printed PCL porous scaffolds containing MWCNTs and HA [21]. The addition of HA and MWCNTs has been shown to enhance mechanical properties and cellular activities, facilitating cell proliferation, differentiation, and mineralization [21]. Moreover, the PCL/HA/MWCNT scaffolds exhibit a structure similar to native bone, mimicking it from the nanoscale to macroscale levels, which suggests their potential application in bone tissue engineering [21]. Additionally, the incorporation of HA has been found to improve the adhesion and viability of loaded mesenchymal stem cells (MSCs). The interconnected porous structures of these hybrid scaffolds provide an ideal environment for the adhesion and proliferation of loaded MSCs, thanks to the ample structural space that enables the efficient exchange of nutrients and metabolic wastes [19]. This favorable environment also promotes the osteogenic differentiation of MSCs, thereby contributing to bone regeneration [18,19]. The 3D architecture of the hybrid scaffolds plays a crucial role in providing a microenvironment conducive to the osteogenic differentiation of MSCs [20].

The basic concept of bone tissue regeneration suggests that successful bone repair is achieved by creating a microenvironment that incorporates osteoinductive factors, osteogenic cells, and an appropriate scaffold in an optimal environment [22]. This study aimed to investigate the efficacy of rabbit bone marrow-derived mesenchymal stem cell (rBMSC)-laden nanomaterial scaffolds in the repair of critical-size segmental bone defects in rabbit radius. The primary focus is to assess the regenerative potential of MSCs when incorporated into nanomaterial scaffolds and to evaluate the overall bone healing outcomes.

## 2. Materials and Method

### 2.1. Experimental Animals

A randomized controlled study was conducted on thirty-six matured adult New Zealand white rabbits, comprising both sexes (1:1), with an average weight of approximately 2.60 ± 0.46 kg and an age range of 6 to 12 months. The rabbits were provided with a standard diet, had ad libitum water, and adhered to a 12 h dark/light cycle. Individual housing in cages was maintained for all animals, and a two-week acclimatization period was observed before the initiation of the study. The animal experiments strictly adhered to the ARRIVE (Animal Research: Reporting of In Vivo Experiments) guidelines and were carried out following the U.K. Animals (Scientific Procedures) Act, 1986, and associated guidelines, as well as the EU Directive 2010/63/EU for animal experiments.

### 2.2. Isolation, Culture, and Expansion of Bone Marrow-Derived Mesenchymal Stem Cells

The rBMSC was isolated, cultured, and expanded as per the standard protocol [23]. Anesthesia was induced in the rabbits using xylazine hydrochloride (6 mg/kg), followed by intramuscular administration of ketamine hydrochloride (60 mg/kg). Bone marrow aspirate was then collected from the lateral iliac crest of the rabbits. The aspirate underwent further processing to isolate mononuclear cells using the density gradient method. The isolated cell pellets were then supplemented with complete growth media comprising 15% fetal bovine serum, 1% antimycotic-antibiotic (100 units/mL of penicillin and 100 µg/mL of streptomycin), and 84% Dulbecco’s Modified Eagle Medium (DMEM). These cell pellets were loaded into sterile T-25 flasks, which were placed in a CO_2_ incubator under specific conditions (37 °C, 5% CO_2_, 85% relative humidity). Over the initial 7 days, cell adherence was monitored every 3 days, with unattached cells removed and fresh media added as needed. Subsequently, media replenishment occurred every 3–4 days once the cells reached 80% confluence. To lift attached cells for further passaging, trypsinization was performed using trypsin (0.25%) EDTA. The lifted cells underwent neutralization and centrifugation to collect suspended cell pellets. Depending on research requirements, cells were expanded and sub-cultured further. Cells from the “P3” population were then utilized for the subsequent in vivo study.

### 2.3. Preparation of Nano-Scaffolds

**Figure 1:**
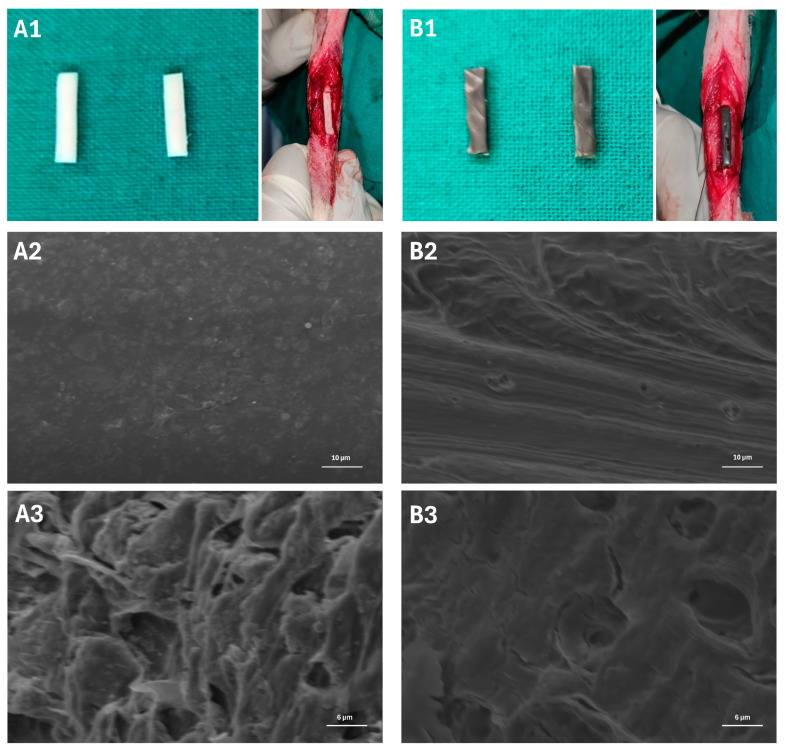
(**A1**) Nanocomposite polycaprolactone (PCL) + hydroxyapatite (HAP) scaffold and (**B1**) nanocomposite PCL + HAP + multiwalled carboxylated carbon nanotube scaffold surgically placed in the critical-sized defect in the radius bone. Scanning electron microscope (SEM) image of PCL + HAP scaffold (**A2**: 1000× and **A3**: 2000×). SEM image of PCL + HAP + MWCNT-COOH scaffold (**B2**: 1000× and **B3**: 2000×).

The nano scaffolds of PCL + HAP and PCL + HAP + MWCNT-COOH with the composition 4 wt% HAP/PCL and 4 wt% HAP + 0.1 wt% MWCNT-COOH/PCL, respectively, were prepared using electrospinning process in Indian Institute of Technology (IIT) at Roorkee, Uttar Pradesh (Figure 1). The scaffolds had dimensions of 15 mm (L) × 0.3 mm (W) × 0.3 mm (H). The scaffolds were fixed at 4 °C for 2 h in a 2.5% glutaraldehyde solution. Subsequently, following a PBS wash, the samples underwent dehydration through a series of graded alcohol concentrations (30%, 50%, 70%, 80%, 90%, and 100%). Supercritical drying was then conducted using hexamethyldisilazane (HMDS) for 10 min. Following air drying and gold sputtering, the specimens were examined under a scanning electron microscope, capturing images at different magnifications [24].

### 2.4. Stem Cell Loading into Nano-Biomaterial Constructs

The nanocomposite scaffold underwent irradiation (type C UV) before a 24 h incubation at 37 °C in a phosphate-buffered solution containing 50 µM/mL fibronectin. In a 2.5 mL centrifuge tube, the fibronectin-pretreated scaffold was positioned at the bottom. Subsequently, 2 mL of complete media containing rBMSC (5 × 10^5^ cells) was added, and cells were loaded onto the scaffold through a centrifugation process consisting of three cycles. Each cycle involved centrifuging the tube at 500 rpm for 2 min, with a 1 min pause between cycles. Following the centrifugation cycles, the cell-seeded scaffold was transferred for in vivo applications in complete media.

### 2.5. Rabbit Radius Critical-Sized Defect Creation

The anterio-medial approach was employed to access the forelimb radius bone following standard surgical procedures [25,26]. After preparing the surgical sites by shaving and disinfecting the areas, skin incisions were made over the radius bones. Soft tissue dissection followed, exposing the underlying bones while minimizing damage to surrounding structures. A 15 mm osteo-periosteal cortical bone segment in the mid-diaphysis of the radius was excised using an oscillating bone saw, with cold normal saline applied during saw motion to prevent thermal necrosis at the fracture ends [25,26]. After creating the defects, the surgical sites were irrigated to remove debris, and any bleeding was controlled. The soft tissues were then carefully repositioned, and the skin incisions were closed using surgical sutures.

### 2.6. Experimental Design and Treatment Protocol

The segmental defect was treated according to the protocol given in Table 1. The composite scaffold, tailored to the respective group, with or without BMP-2 and/or rBMSC, was implanted into the bone defect, excluding group A, which underwent external splinting alone. The surgical site underwent muscle, subcutaneous tissue, and skin closure using 3-0 polyglactin 910 sutures. Initially, the surgical site was immobilized with splinting for one week, and antiseptic dressing was maintained until complete skin healing. In group D, 0.4 µg/mL of BMP-2 was administered via transcutaneous route at the scaffold implantation site on the postoperative days 30, 45, and 60. Group E animals received three million rBMSC (P3) at the scaffold implantation site on postoperative days 7, 14, and 21. Animals in group F received both three million rBMSC (P3) at the scaffold implantation site on days 7, 14, and 21 and 0.4 µg/mL BMP-2 on days 30, 45, and 60. At 90 days post-implantation, the animals were euthanized following standard guidelines [27], and the experimental bone was harvested for further investigations [28].

### 2.7. Clinical Observations

Vital parameters, including heart rate (beats/minute), respiratory rate (breaths/minute), and rectal temperature (°F), were recorded on the postoperative days 0, 1, 2, 3, 4, 5, 6, and 7 in a controlled lab environment. Clinical observations, such as pain, swelling, and exudation at the ostectomy site, were documented on the postoperative days 0, 1, 2, 3, 4, 5, 6, 7, 8, 9, 10, 30, 60, and 90, utilizing a standard scoring system (Supplementary Tables S1 and S2) [29]. Similarly, the lameness scoring was carried out one day before and on postoperative days 1, 15, 30, 45, 60, and 90, utilizing a standard scoring system [30].

### 2.8. Gait and Mobility Analysis

The footprints of the experimental animals were analyzed using the standard method to assess various parameters related to gait and mobility (right forefoot stride length, left forefoot stride length, right hind foot stride length, left hin foot stride length, forefoot step length, and drag mark length). The animals were allowed to walk freely after applying dye to all four paws, and footprints were recorded on the day before (pre) and postoperative days 1, 30, 60, and 90 for all groups following the protocol by Wilkinson et al. (1995) [31]. The parameters measured included the stride lengths of the right forelimb, left forelimb, right hind limb, and left hind limb, which represent the linear distances (cm) between successive points of limb-to-floor contact during both the stance and swing phases. Additionally, the forelimb step length, representing the linear distance (cm) between heel strikes on contralateral forelimbs, was measured. The analysis also included the drag mark length, which measures the length of drag staining from the most cranial foot nail floor contact during stance to the next floor contact of the same foot during normal walking. The drag mark, which is observed in the swing phase, may exhibit curvilinear characteristics, and its length was measured using a thread and extrapolated over a measuring tape.

### 2.9. Serum Biochemical Parameters

Biochemical parameter analysis included the estimation of blood glucose (mg/dL, serum total protein (g/dL), alkaline phosphatase (ALP) (IU/L), phosphorus (mg/dL), and ionized calcium (mg/dL) at different time points in this study. Blood glucose levels were determined from ear tip samples using a glucometer strip on day 0 (preoperatively) and on the postoperative days 30, 60, and 90 across different groups. Serum total protein, ALP, phosphorus, and ionized calcium levels were estimated on day 0 (preoperatively) and the postoperative days 30, 60, and 90 in various groups.

### 2.10. Radiographic Analysis

Radiographic analysis was performed using a computed radiography system (Medion MX 32 HF, Medion Healthcare Pvt. Ltd., Maharashtra, India) with exposure factors set at 44 kV and 2.2 mA, maintaining a constant X-ray to object to film distance of 171 cm. A standardized medio-lateral view radiograph was taken on day 0 (pre- and postoperative) and subsequently at 30, 60, and 90 days postoperatively. The assessment involved evaluating osseous union, if present at the host bone–implant interface, observing osseous callus formation over the implant, and identifying any osteogenic reaction. The scoring system used for evaluation was a slightly modified version of the one adapted from Yang et al. (1994) (Supplementary Table S3) [32].

### 2.11. Scanning Electron Microscopic (SEM) Analysis

The SEM examination was employed to investigate the microstructure and surface characteristics of the healed bone tissue at high magnification and resolution. The bone fixation process involved immersing the sample (*n* = 1) from each group in 2.5% glutaraldehyde for 72 h, followed by 1% osmium oxide for 24 h. Subsequently, the samples underwent dehydration in graded alcohol and drying. Ultra-dehydration was achieved using hexamethyldisilazane before mounting the samples onto SEM stubs using carbon tape. The samples were then coated with a thin layer of gold to enhance conductivity by sputter coating (10 mA, 90 s, 2.0 × 10^−2^ torr) (G20 Ion Sputter Coater, GSEM, Suwon-si, Gyeonggi-do, Republic of Korea). The processed bone samples were then analyzed at a 25 kV accelerating voltage using a scanning electron microscope (Cube II, EMCrafts, Gwangju-si, Gyeonggi-do, Republic of Korea) [33].

### 2.12. Histopathological Analysis

At 90 days post-implantation, the animals underwent euthanasia through intracardiac overdosing of thiopental sodium at a dose of 80 mg per kg body weight. The collected bone samples (*n* = 6) from each group were fixed and immersed in a decalcifying solution until they were ready for microtome dissection, employing Gooding and Stewart’s fluid [34]. After microtome cutting, the tissues were mounted on poly-l-lysine-coated slides and stained with hematoxylin–eosin (H&E) and special Masson’s trichrome stain for collagen tissue evaluation. The stained slides were assessed using a modified scoring system based on the criteria reported by Lane and Sandhu (1987) and Heiple et al. (1987) (Supplementary Table S4) [35,36].

### 2.13. Statistical Analysis

Quantitative data from different groups and various time intervals, assumed to follow a normal distribution, underwent analysis using repeated measures ANOVA with post hoc Tukey testing (Statistical Program for Social Sciences Version 20.0 software, IBM Corp, Armonk, NY, USA). Non-normally distributed or qualitative data across different groups at specific time intervals were assessed utilizing the Kruskal–Wallis test. For comparisons within a group at different time intervals or for non-normally distributed data, the Wilcoxon test was employed. Two-sample comparisons were conducted using the *t*-test. The results are presented in the mean ± SD format, and statistical significance was set at *p* < 0.05.

## 3. Results

### 3.1. Surface Morphology of the Scaffold

The surface morphology of the PCL + HAP and PCL + HAP + MWCNT-COOH scaffolds is illustrated in Figure 1. In both scaffolds, the structural components were uniformly distributed with the presence of minute pores. The incorporation of hydroxyapatite contributed to the rough surfaces observed in both scaffolds. Despite the irregular surfaces in both, the PCL + HAP scaffold appeared comparatively smoother than the PCL + HAP + MWCNT-COOH scaffold at higher magnification. Furthermore, a distinct polymeric pattern was observed in PCL + HAP and PCL + HAP + MWCNT-COOH. The SEM images revealed subtle differences between the scaffolds at higher magnification, possibly attributed to the very low concentration of MWCNT-COOH.

### 3.2. Clinical Findings

No significant differences were observed in the heart rate, respiratory rate, and rectal temperature between groups or within individual groups at various intervals compared to pre-trial values (Supplementary Tables S5–S7). All the values remained within the normal range throughout the observation period. Feed and water intake slightly decreased in groups A, B, C, D, E, and F on the day of surgery and up to the 5th postoperative day in all groups, except in group A, where the reduction extended up to 7–8 days.

**Figure 2:**
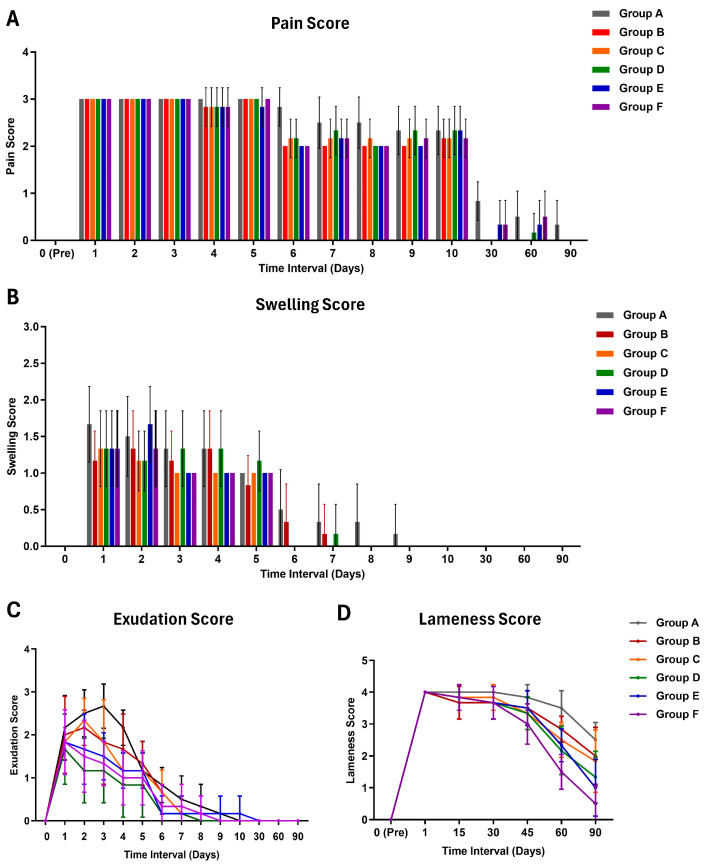
Histogram (mean ± SD) showing (**A**) pain score and (**B**) swelling score of various treatment groups at different time intervals. Line graph (mean ± SD) showing (**C**) exudation score and (**D**) lameness score of various treatment groups at different time intervals.

The pain scores showed no significant differences among groups at pre- and 1, 2, 3, 4, and 5 days postoperatively (Figure 2) (Supplementary Table S8). On the 6th day, group A exhibited significantly higher (*p* < 0.05) pain scores compared to other groups. Within-group comparisons indicated that group A experienced pain up to 30 days, while other groups showed pain up to the 10th postoperative day. Between-group comparisons in swelling scores showed no significant differences throughout the observation period (Supplementary Table S9). However, within-group comparisons revealed significant swelling up to the 5th postoperative period in all groups. Exudation scores demonstrated no significant differences among groups before and on the 1st day after surgery (Supplementary Table S10). However, on days 2, 3, 4, and 6 after surgery, group A exhibited significantly higher exudation scores. Within-group comparisons revealed that all groups showed significantly increased exudation up to the 5th postoperative day.

Lameness scores showed no significant differences between groups during pre-, day 1, and 30th day of observation (Figure 2) (Supplementary Table S11). On the 45th day, groups A and F exhibited significantly higher lameness scores, while groups B, C, and D had intermediary scores. Within-group comparisons revealed that groups A, B, C, and D had significant lameness throughout the observation period, while groups E and F showed no significant lameness on the 90th day. The maximum lameness score was reached on the first day after the operation, and in group A, it persisted in a static phase up to the 30th day of observation.

### 3.3. Impact on Gait and Mobility

No significant differences were observed both between and within groups across various time intervals throughout the observation period for left hind foot stride, right hind foot stride, and left forefoot stride (Supplementary Tables S12–S17). For the right forefoot stride, there was no significant difference among the groups up to the 45th day of observation, though all groups recorded their lowest mean values at this point. By the 60th day, group A exhibited significantly (*p* < 0.05) shorter strides, followed by groups B, E, and F. Conversely, groups C and D showed significantly (*p* < 0.05) longer right forefoot stride lengths on the 60th day. On the 90th day, group A had a significantly (*p* < 0.05) lower right forefoot stride length compared to the other groups. Throughout the observation period, all groups displayed decreased right forefoot stride length compared to pre-values (Supplementary Tables S12–S17). In within-group comparisons, groups A and B consistently showed significantly (*p* < 0.05) lower right forefoot stride lengths from the 15th day of observation to the end of the period. Groups C and D exhibited significantly (*p* < 0.05) higher stride lengths, with groups D and F having non-significantly higher lengths on the 1st postoperative day.

For forefoot step length, the 1st postoperative day comparison revealed that group A had significantly (*p* < 0.05) shorter step length than groups C, D, E, and F, with group B having an intermediary value. From the 15th to the 45th postoperative day, group A consistently exhibited significantly (*p* < 0.05) shorter forefoot step length than the other groups (Supplementary Tables S12–S17). On the 60th day, group A had significantly (*p* < 0.05) lower step length, and group D had an intermediary value between group A and the remaining groups. By the 90th day, group E had the significantly highest, and group A had the lowest forefoot step length, with the remaining groups falling between group D and Group E. In within-group comparisons, group A showed significantly (*p* < 0.05) lower forefoot step length throughout the observation period, and group C displayed significantly (*p* < 0.05) lower step length up to the 45th day. In drag mark analysis, significant differences were observed between experimental groups from the 1st to the 90th postoperative day, with group A consistently displaying significantly (*p* < 0.05) more drag marks than the other groups (Supplementary Tables S12–S17). However, from the 15th day onwards until the end of the observation period, these groups did not show any drag marks. In within-group comparisons, group A displayed significant (*p* < 0.05) drag marks until the 60th day of observation, with the maximum observed on the 1st postoperative day.

### 3.4. Serum Biochemical Changes

No significant differences were observed in the blood glucose level between the groups, and values remained consistent across various intervals within each group when compared to their respective pre-trial values (Supplementary Table S18). Throughout the observation period, blood glucose levels were found to be within the normal range. There were no significant differences in the serum total protein value between the groups or within each group at different intervals, except for group A (Supplementary Table S19). Notably, on the 60th and 90th days post-surgery, group A displayed a significant decrease in total protein levels compared to the pre-trial values.

**Figure 3:**
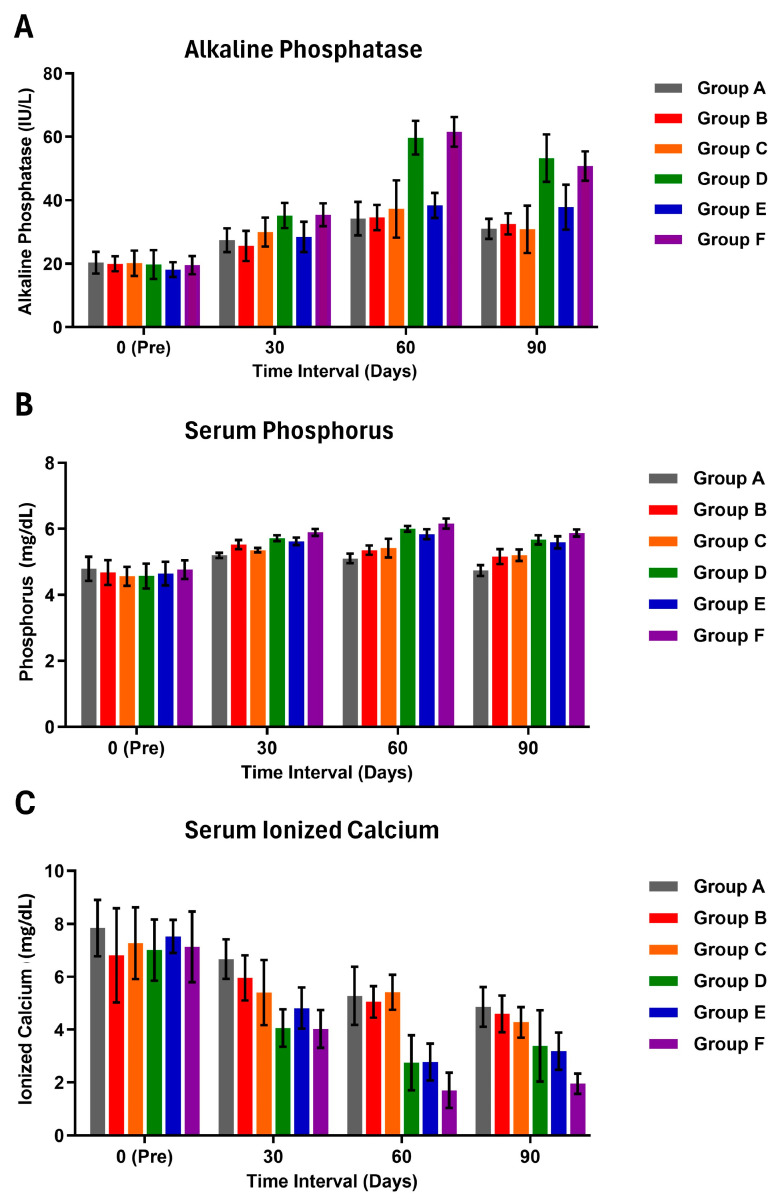
Histogram (mean ± SD) showing (**A**) serum alkaline phosphatase (IU/L), (**B**) serum phosphorus concentration (mg/dL), and (**C**) serum ionized calcium (mg/dL) of various treatment groups at different time intervals.

In serum ALP levels, no significant differences were observed between groups on the pre-surgery 0th day (Figure 3). On the 30th day, groups A, B, and E exhibited significantly (*p* < 0.05) lower ALP activity than groups D and F (Supplementary Table S20). Group C showed no significant difference with either increased ALP activity in groups D and F or with groups A, B, and E. On the 60th day, all groups reached their maximum ALP activity compared to other time intervals within their respective groups. Furthermore, on the 60th and 90th days, intergroup comparisons indicated that groups D and F had significantly higher ALP activity than the other groups.

In serum phosphorus levels, no significant differences were noted between groups on the pre-surgery 0th day (Figure 3). On the 30th day, groups F and D showed significantly higher values, while groups A and C had lower values compared to the others (Supplementary Table S21). Maximum phosphorus levels for groups A and B were reached on the 30th day, and for groups C, D, E, and F, they were reached on the 60th day. Specifically, on the 60th day, group F exhibited significantly (*p* < 0.05) higher phosphorus levels than groups D and F. On the 90th day, group A had significantly lower phosphorus levels than other groups, with no significant difference between groups B and C (Supplementary Table S21). Both groups B and C had significantly lower values than groups D, E, and F. Except for group A, all other groups displayed increased phosphorus values compared to their respective pre-values. Within-group comparisons indicated that group A had a significant (*p* < 0.05) increase in phosphorus on the 30th day, while groups B, C, D, E, and F showed significantly higher phosphorus levels on the 30th, 60th, and 90th days compared to pre-surgery 0th day levels (Supplementary Table S21).

No significant differences were observed in the serum ionized calcium value between the groups on the pre-surgery 0th day (Figure 3). On the 30th day, no significant differences were found among groups A, B, and C, while groups D, E, and F exhibited significantly lower ionized calcium than group A (Supplementary Table S22). Group C showed no significant difference from other groups, with group A having higher ionized calcium and group F displaying the lowest levels. On the 60th day, groups A, B, and C had significantly (*p* < 0.05) higher ionized calcium than groups D, E, and F (Supplementary Table S22). Groups D, E, and F had lower ionized calcium levels than others on the 60th day. By the 90th day, group F had the lowest serum ionized calcium, significantly (*p* < 0.05) lower than groups A, B, and C, but not significantly lower than groups D and E. Overall, there was a significant decrease in serum ionized calcium in all groups at every time interval compared to their respective pre-values, except for group A on the 30th day and group B on the 30th and 60th days, which showed significantly lower values (Supplementary Table S22).

### 3.5. Radiographic Findings

**Figure 4:**
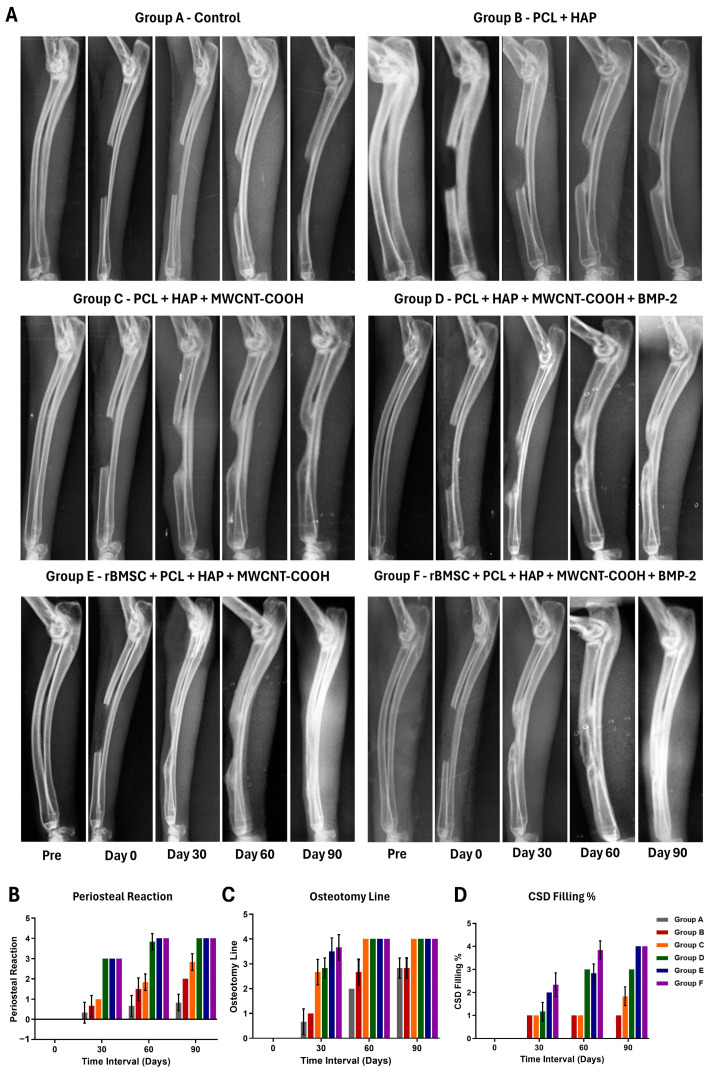
(**A**) Medio-lateral radiographs showing healing status of different groups at various time intervals (pre, day 0, day 30, day 60, and day 90). Histogram (mean ± SD) showing radiographic (**B**) periosteal reaction score, (**C**) osteotomy line, and (**D**) critical-size defect (CSD) filling % score of various treatment groups at different time intervals.

In the radiographic assessment of periosteal reaction, initial comparisons among the groups demonstrated no significant differences on day 0 (Figure 4). However, by the 30th day, groups D, E, and F exhibited a notably higher reaction compared to groups A, B, and C, with statistical significance (*p* < 0.05) (Supplementary Table S23). Furthermore, a significant difference in periosteal reaction was observed between groups D and E on the 30th day. On the 60th day, group B displayed a significantly lower periosteal reaction than the other groups, while groups B and C did not show a significant difference between them (Supplementary Table S23). In contrast, groups D, E, and F continued to demonstrate a significant periosteal reaction. Similar trends persisted on the 90th day, with significant radiographic differences observed between groups D and E. However, when compared to group F, groups D and E did not exhibit a statistically significant difference (Supplementary Table S23).

Comparing osteotomy line scores between groups, it was found that throughout the study period, there was no significant difference between groups A and B (Supplementary Table S24). However, on day 60, group B exhibited a significant (*p* < 0.05) improvement in the osteotomy line score. On day 30, groups E and F demonstrated significantly better scores than groups C and D, with group D having a higher mean value, although not statistically significant. On days 60 and 90, no significant differences were observed among groups C, D, E, and F (Supplementary Table S24).

The assessment of critical-size defect filling scores in various experimental groups over different observation periods revealed distinct outcomes (Supplementary Table S25). Throughout the experimental period, we observed significant differences in the critical-size defect filling scores among the treatment groups. Notably, groups B, C, D, E, and F exhibited significant improvements in defect filling compared to the control group (Group A) at all time points (days 30, 60, and 90) (Supplementary Table S25). By Day 90, Groups E and F demonstrated the highest defect filling scores, followed by group D, signifying robust and sustained bone regeneration over time.

In the control group (Group A), no significant new bone formation was observed, resulting in an unaltered critical-size defect and non-union (Figure 4). In group B, where PCL + HAP was applied, osteogenesis was initiated on day 30 and became pronounced on days 60 and 90. While the new bone formation connected with the adjacent ulna at both ends of the defect, it could not completely fill the cortical defect, though it showed improvement compared to group A. Group C, employing PCL + HAP + MWCNT-COOH nano scaffold, exhibited mild to moderate bone formation, surpassing both groups A and B. By day 30, new bone emerged from both defect ends; on day 60, a more pronounced reaction reduced the defect size, connecting with the adjacent ulna; and on day 90, the defect size further decreased, with both cut ends tightly bridged to the adjacent ulna (Figure 4). In group D, incorporating BMP-2 into the PCL + HAP + MWCNT-COOH nano scaffold resulted in superior new bone formation compared to groups A, B, and C. By day 60, the defect was filled with new bone, and by day 90, further reduction in defect size was evident, with new bone bridging with the adjacent ulna. Group E, where rBMSC was applied in the nano scaffold, exhibited robust osteogenesis/new bone formation. On day 30, new bone formation bridged with the ulna, and by day 60, the critical-size defect was almost entirely filled. By day 90, new bone completely occupied the defect, making it challenging to identify the defect location, and periosteal continuity was established. In group F, where both BMP-2 and rBMSC were applied in the nano scaffold, excellent new bone formation was observed (Figure 4). By day 30, new bone formation emerged from both cut ends, bridging the defect gap. By day 60, the defect gap was completely filled with new bone, connecting with both cut ends, and periosteal continuity developed. On day 90, extensive new bone formation obliterated the bone defect, and periosteal continuity was well-established. The overall new bone formation was highest in group F, followed by groups E, D, C, and B. No new bone formation was noticed in the control group (Group A).

### 3.6. Scanning Electron Microscopy

**Figure 5:**
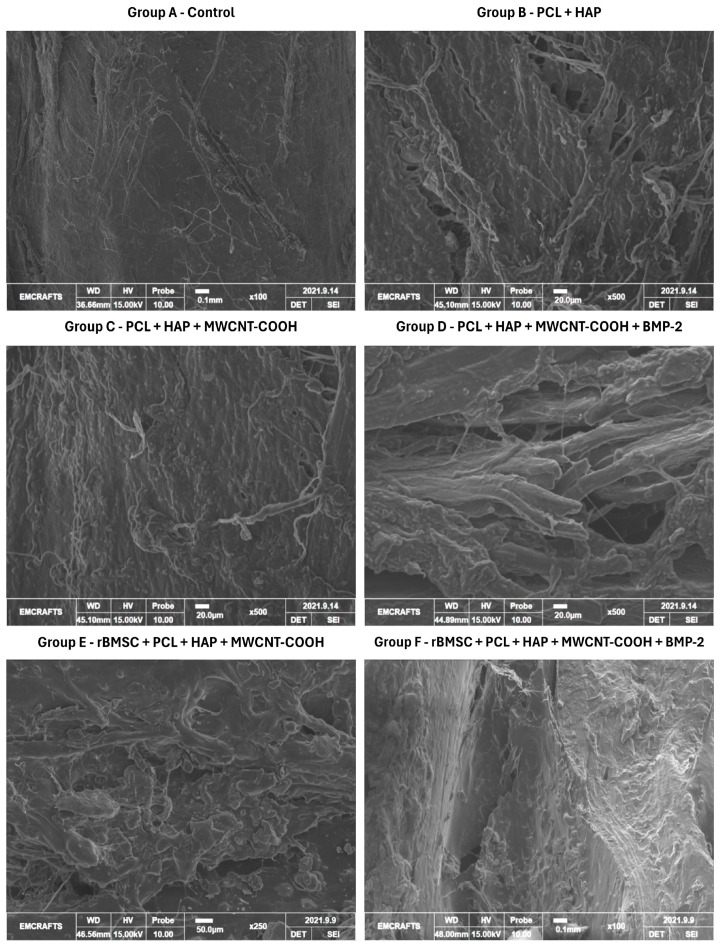
Scanning electron microscope (SEM) images depicting healed bone tissue samples from different groups on day 90.

SEM analysis of the mid-diaphyseal radius bone in group A revealed a predominance of collagen fibrils. Additionally, examination of the defect site demonstrated filling primarily with fibrous tissue, with a notable absence of osseous tissue. These observations confirm it to be a healed critical-size defect characterized by a cessation of osteogenesis (Figure 5). Group B exhibited significant morphological changes, including fissures and disintegrated bone architecture. In group C, there was notable restoration of bone morphology, characterized by reduced pore formation, improved lamellar bone architecture, and increased compactness compared to groups A and B. Group D showed increased lamellar bone formation and a larger marrow space. Group E demonstrated thickened mineral deposition over a lamellar structure, nearly filled bone defect with newly developed osseous tissue, neovascularization, abundant bone cells, and reduced marrow spaces. Finally, group F exhibited increased compactness, a smooth surface, and reduced porosity, indicating superior bone regeneration. Furthermore, group F had better compactness and new bone formation than groups D and E.

### 3.7. Histopathological Findings

**Figure 6:**
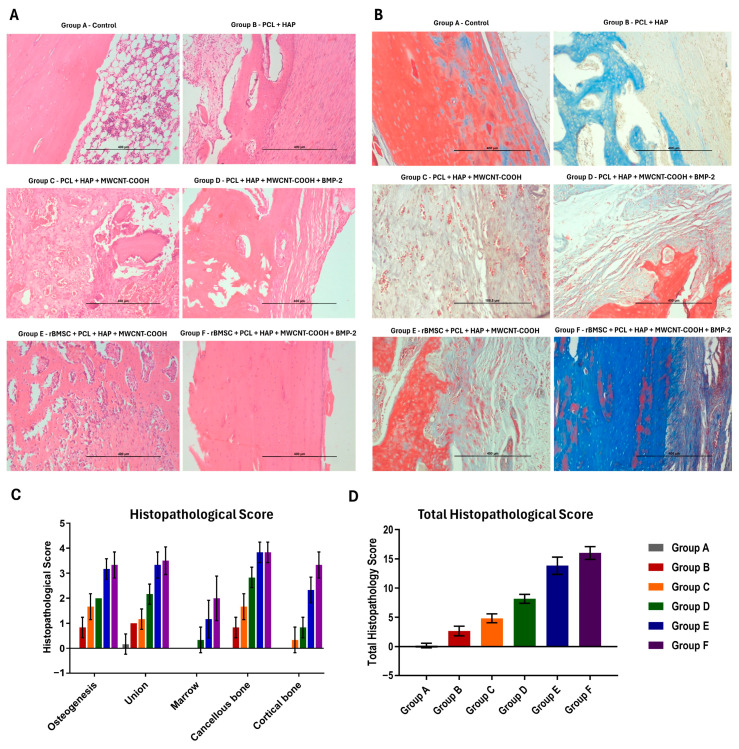
(**A**) Histopathological images depicting healed bone tissue samples from different groups on day 90, stained with hematoxylin and eosin (H&E) for microscopic analysis (scale bar: 400 µm). (**B**) Histopathological images depicting healed bone tissue samples from different groups on day 90, stained with Masson’s trichrome for microscopic analysis (scale bar: 400 µm). Histogram (mean ± SD) showing the (**C**) histopathological score and (**D**) total histopathological score of various treatment groups on day 90.

In the transverse sections of group A samples stained with H&E, the cut ends exhibited a characteristic blunted appearance, with fibrous tissue protruding at the edges (Figure 6). However, minimal collagen fiber deposition was observed in the Masson’s trichrome staining. The defect site was predominantly filled with fibrous tissue, indicating a lack of proper osteogenic and osteoconductive processes essential for bone healing. Comparison with the adjacent ulna, which displayed intact bone architecture, underscored the absence of new bone deposition in the defective radius. This observation suggests a potential risk of non-union fracture progression in the affected area (Supplementary Table S26).

In group B, H&E staining of transverse sections revealed abundant yet loose collagen fibers in connective tissue at the cut end interface. Sections stained with Masson’s trichrome revealed the presence of immature connective tissue in the critical-size defect region, characterized by faintly stained blue fibers. Additionally, a wide marrow space was observed in the same area, indicating incomplete bone regeneration. Small vascular cells were observed within the faintly blue-stained connective tissue structures. Although subtle bone formation was noted, it had yet to mature and deposit calcium super saturation crystals.

Group C displayed densely packed fibroblasts with haphazardly patterned collagen fibers. The corresponding bone section, stained with H&E, exhibited extensive vascular changes, severe congestion, bluish-stained calcium deposits, and infiltration of inflammatory cells in the critical-size defect region. The majority of the spaces were filled with connective tissues with extensive vascular structure proliferation, whereas a wider-spaced, smaller vascular cell population was observed in group B. Calcium deposition exhibited a distinct pattern within the collagen connective tissue matrix. Despite the absence of scaffold remnants, the faster resorption led to angiogenesis and discrete calcium mineral super saturation in the newly embedded connective tissue islands.

For group D, H&E staining revealed a callus-bridged bone morphology, bone spicules, and evident mineralization areas in the endosteal callus formation. The corresponding bone section, stained with Masson’s trichrome, depicted normal bone surrounded by immature collagen fibers, demonstrating the healing process. Furthermore, in the endosteal region, there was a reduction in space and an increase in spicule formation, which was supported by osteogenic cells. A higher density of osteoblasts was observed in conjunction with the immature connective tissue fibers. Within the matured region, osteocytes were observed along with spicule formation extending from the endosteal region towards the critical-size defect area. Additionally, no evidence of scaffold remnants was observed in this group.

In Group E, H&E staining of transverse sections showcased completely healed bone morphology, with evident blood vessel canals at the cortex area and clear bone marrow surrounded by dense fibrous tissue. Masson’s trichrome staining depicted a dense cortex, soft bone marrow, and normal bone characteristics. The bone section stained with H&E displayed thick osteoid tissue deposition and connective tissue formation. Masson’s trichrome staining indicated collagen fiber formation during the healing process, with osteoblasts lining the endosteal region and the presence of osteocytes.

Group F exhibited completely healed remodeling bone morphology in H&E-stained transverse sections, with endosteal bone marrow formation and calcium depository areas. Masson’s trichrome staining revealed a dense cortex and loose areolar bone marrow. The H&E-stained bone section illustrated complete osteoid formation along with osteocytes, resembling normal bone histo-architecture. Corresponding Masson’s trichrome staining confirmed matured collagen fiber formation. The dense cortical region showed well-packed osteocytes within the connective tissue matrix, suggesting a stage between osteogenesis and remodeling, where new bone formation and old bone resorption depended on the load upon the bone.

## 4. Discussion

The body’s natural ability to regenerate bone tissue has its limitations, especially when it comes to larger defects that exceed its inherent capacity. Critical-size defects are substantial gaps in bone structure that cannot heal on their own and typically require surgical intervention or the application of regenerative therapies to prompt bone repair [37]. Untreated critical-size defects can lead to serious complications, including non-union, delayed union, or malunion, which may result in chronic pain, instability, and deformity [38]. Conventional treatment methods like bone grafting may prove inadequate for addressing such extensive defects, necessitating the exploration of alternative approaches such as tissue engineering and regenerative medicine to achieve successful outcomes [39]. Addressing critical-size defects in bone solely with implants presents several challenges [40,41,42]. Firstly, implants may not offer adequate support or stimulation for optimal bone regeneration, potentially leading to implant failure due to issues like inadequate osseointegration, instability, or mechanical stress [40,41]. In cases of critical-size defects, limited bone volume or poor bone quality can further impede proper integration with the implant surface [40]. Moreover, implants alone may lack the necessary biological cues or scaffolding to effectively promote tissue regeneration, highlighting the need for complementary approaches in addressing such complex bone defects [40,41,42].

To address the challenges posed by critical-size defects, various therapeutic strategies are employed at preclinical and clinical experimental levels. Biomaterials, both natural and synthetic, serve as fillers at bone loss sites, often combined with growth factors, cellular therapies, and compounds conducive to niche formation. The documented use of various biomaterials includes grafts, Ilizarov distraction osteogenesis, cellular transplantation (MSCs from different sources), mineral substitutes (bioceramics), collagenous substitutes (natural collagen, polymers, and hydrogels), and composites containing all bone tissue-forming constituents [43]. Despite the array of strategies available, considerations such as fracture location, geometry, implant characteristics, and bone environment status must be factored in [44]. Common treatments for critical-size defects include bone grafting, distraction osteogenesis, and the induced membrane technique. Autografting is regarded as the gold standard technique, but its use is limited by factors such as a lack or limited source of autogenous tissue, donor site complications (hernia, chronic pain, surgical risk), and the restricted potential of collected tissue in terms of anatomy, physiology, and cognition processes [43,45]. Allogeneic bone can be an alternative, addressing some limitations but introducing ethical concerns, limited vascular support, and the possibility of immune rejection. Xenogeneic options face challenges related to strong immune reactions and ethical disputes. Therefore, alternative bioimplants are essential to overcome supply limitations and enhance osteogenic support beyond existing methods.

Biomaterials, including bioactive ceramics and biopolymers, play crucial roles in bone regeneration by providing structural support, promoting cellular attachment and proliferation, and facilitating the formation of new bone tissue [46]. Bioactive ceramics, such as HA and TCP, have excellent biocompatibility and bioactivity, making them ideal for bone regeneration. These ceramics possess chemical compositions similar to the mineral phase of natural bone, allowing for direct bonding with host bone tissue [47]. When implanted, bioactive ceramics can stimulate osteogenic cell activity and promote the deposition of new bone matrix, leading to enhanced bone regeneration. Additionally, they can act as scaffolds for cell attachment and proliferation, providing a conducive environment for bone formation [46,47]. Biopolymers, on the other hand, offer versatility and tunability in terms of mechanical properties and degradation rates, making them suitable for various bone regeneration applications [46,48]. Biopolymers like polylactic acid (PLA), polyglycolic acid (PGA), and their copolymer PLGA are widely used in bone tissue engineering due to their biodegradability and ability to support cell growth [48]. These polymers can be fabricated into porous scaffolds with interconnected pore structures, mimicking the extracellular matrix of natural bone and providing a substrate for cell attachment, proliferation, and differentiation [46,48].

One of the key advantages of polymers is their versatility, as they can be tailored to possess a wide range of mechanical properties, degradation rates, and surface characteristics to suit specific requirements [49]. Biodegradability is another significant advantage, with many polymers being designed to gradually degrade and be absorbed by the body, leaving behind regenerated tissue [49,50]. Additionally, polymers are generally biocompatible and well-tolerated by the body, reducing the risk of adverse reactions or rejection. They can be easily processed into various forms, such as films, fibers, or porous scaffolds, providing structural support and promoting cell attachment, proliferation, and differentiation [49,50,51]. Moreover, polymers can be functionalized for drug delivery, encapsulating bioactive molecules or growth factors to enhance tissue regeneration while minimizing systemic side effects [49,50]. However, challenges such as controlling degradation rates, ensuring sufficient mechanical strength, and addressing potential inflammatory responses must be carefully addressed to optimize the efficacy of polymer-based biomaterials in bone regeneration studies and clinical applications [51].

Electrospinning is a versatile and widely used technique in the field of tissue engineering, particularly for creating scaffolds for bone tissue regeneration [52,53,54,55,56]. The process involves the use of an electric field to draw a charged polymer solution or melt it into ultrafine fibers, which are then deposited onto a collector to form a non-woven mesh-like structure [53]. The setup typically consists of a syringe containing the polymer solution or melt, a high-voltage power supply, and a grounded collector. During electrospinning, when a high voltage is applied to the polymer solution, it forms a charged droplet at the tip of the syringe needle [53,54]. The electrostatic repulsion between the charges overcomes the surface tension of the droplet, causing a thin jet of polymer solution to elongate and rapidly solidify as it travels toward the collector due to solvent evaporation or cooling [53,54]. This results in the formation of nanoscale or microscale fibers that accumulate on the collector to create a three-dimensional scaffold [53]. The significance of electrospinning in creating scaffolds for bone tissue engineering lies in its ability to produce biomimetic structures with a high surface area-to-volume ratio, interconnected porosity, and tunable mechanical properties [55,56]. These electrospun scaffolds closely mimic the ECM of natural bone, providing an ideal microenvironment for cell attachment, proliferation, and differentiation [55,56]. Additionally, the nanofibrous architecture of electrospun scaffolds can enhance nutrient diffusion, waste removal, and the exchange of signaling molecules, promoting tissue ingrowth and vascularization. This versatility and scalability make electrospinning a valuable tool for fabricating customized scaffolds tailored to specific applications in bone tissue engineering, including bone defect repair, bone graft substitutes, and drug delivery systems [52,53,54,55,56].

The SEM images revealed only a subtle difference between the scaffolds (PCL + HAP and PCL + HAP + MWCNT-COOH) at higher magnification in our study, possibly attributed to the very low concentration of MWCNT-COOH. The composition of nano scaffolds, specifically the incorporation of different materials such as PCL, HAP, and MWCNT-COOH, significantly influences their surface morphology [57]. In scaffolds composed of PCL alone, the surface morphology typically consists of smooth and uniform nanofibers, as PCL is known for its ability to form well-defined fibers during electrospinning [57]. However, the addition of HAP nanoparticles to PCL results in the formation of a rougher surface with irregularities and nanoparticle agglomerates dispersed throughout the scaffold matrix [58]. Furthermore, when MWCNT-COOH is incorporated into the PCL + HAP scaffold, it can further alter the surface morphology [57,58,59]. The presence of MWCNTs, with their inherent tubular structure and functionalized carboxylic acid groups, can lead to the formation of hierarchical structures on the scaffold surface [57,58,59]. Additionally, the interaction between PCL, HAP, and MWCNT-COOH may influence the alignment and orientation of nanofibers, leading to changes in fiber density and alignment on the scaffold surface [57,58,59]. Overall, the composition of nano scaffolds, including the types and concentrations of materials used, plays a crucial role in determining their surface morphology [57].

Serum ALP activity peaked at 60 days in all groups, indicating the initiation of anabolic bone tissue formation. Notably, ALP activity significantly increased on days 60 and 90 in PCL + HAP + MWCNT-COOH + BMP2 (Group D) and PCL + HAP + MWCNT-COOH + rBMSC + BMP2 (Group F), possibly due to BMP2 administration stimulating ALP activity. Group E (PCL + HAP + MWCNT-COOH + rBMSC) showed increased ALP activity, though lower than the BMP-2 administered groups, suggesting the influence of BMP2 administration. The liver and kidney contribute to ALP levels, emphasizing the need for bone-specific ALP estimation for sensitivity [60]. Callus volume correlates directly with ALP activity, and as days progress, phosphorus levels increase. Group D displayed increased phosphorus throughout the observation period, similar to Groups C, D, and E. The control group showed maximum and minimum phosphorus levels on days 30 and 90, respectively. BMP-2 induces calcification via hyperphosphatemia [61]. Although calcium levels reportedly rise sharply around 15–21 days post-surgery, our study observed an increase in phosphorus levels on the 30th day. Overall, increased phosphorus levels were noted in all groups except the control, consistent with previous findings [28]. Ionized calcium levels decreased during fracture healing, likely due to long-term BMP-2 administration favoring higher bone formation. BMP-2 lowers calcium non-significantly in a 60-day bone healing study [28]. Ionized calcium, crucial for bone formation, decreases during progressive phases of bone tissue regeneration [62]. Additionally, BMP-2 administration resulted in lower ionized calcium levels on the 30th and 60th days, suggesting BMP-2’s role in driving calcium from blood to the fracture site for mineralization [63].

Radiographic analysis revealed that Group C (PCL + HAP + MWCNT-COOH) displayed superior periosteal reaction throughout the observation period. While there was initially little difference in periosteal reaction between the control and Group B (PCL + HAP) scaffold on day 30, the PCL + HAP scaffold exhibited increased periosteal reaction in later stages. In the PCL + HAP group, no union was observed between the ends and along the sides of the ulna. Although mild woven bone tissue formation occurred at the ends, it did not significantly differ from Group A. A study using hydroxyapatite alone in a 5 mm defect also reported similar results [64], but the density at the implanted site was higher than our findings. Despite this, our study utilized a scaffold composite with PCL and a smaller quantity of HAP. Group C (PCL + HAP + MWCNT-COOH) scaffold alone also exhibited a notable periosteal reaction on days 30, 60, and 90, albeit significantly less than scaffolds with stem cells alone or with BMP-2. This difference may be attributed to the effect of stem cell differentiation and the synergistic osteoblastic activity of BMP-2 [65]. The increased level of periosteal reaction in BMP-2 was observed similarly to stem cells alone and with BMP-2, aligning with findings in a rabbit distractive bone model with recombinant human BMP-2 [61].

The osteotomy line was notably superior in the group that received stem cells. Both BMP2-doped PCL + HAP + MWCNT-COOH (Group D) and PCL + HAP + MWCNT-COOH (Group C) demonstrated enhanced performance compared to the control and PCL + HAP group. Carbon nanotubes exhibited the ability to stimulate ectopic bone formation even when nanohydroxyapatite did not [66]. The scaffold coated with multiwalled carbon nanotubes expressed the early osteogenesis transcription factor RUNX2, suggesting that PCL + HAP + MWCNT might achieve a favorable osteotomy line score and periosteal reaction [67]. In the control group, gap filling in critical areas was less than 25%, possibly due to the inability to fill the gap within the normal physiological limits of the bone regenerative cascade mechanism. Several limiting factors, including the lack of a scaffold and insufficient growth factors and stem cell stimulatory substances, could contribute to this outcome [68]. On the 60th day, PCL + HAP + MWCNT-COOH + BMP2 + rBMSC (Group F) exhibited the maximum filling of the critical-size defect, potentially attributed to the carbon nanotube-inducible factor on in situ stem cells along with BMP2, promoting bone tissue mineralization. Carbon nanotubes stimulate and guide in situ cells for bone tissue formation due to their bone-tissue compatibility and osteointegration [69]. Additionally, multiwalled CNT-reinforced thermoplastic polymethylmethacrylate (PMMA) demonstrated new bone formation on the surfaces [70]. By the 90th day, both stem cell-received groups exhibited more than 75% filling of the critical-size defect with organized bone, suggesting the differentiation of osteogenic cells under a favorable microenvironment. The rapid skeletal changes in rabbits further support this observation, consistent with earlier findings [71].

Histopathological findings confirmed that the control group exhibited the least osteogenic potential, with the PCL + HAP group showing mineralization that was not promptly evident but an active process of collagen formation. Despite these changes, vasculogenesis was limited in our study, contrasting with the 3D bio-printed PCL alone with electrical stimulation used in a bone regeneration study [72]. The scaffold-alone groups, namely PCL + HAP + MWCNT-COOH, found that the addition of carbon nanotube in combination induces faster osteogenesis. Carbon nanotube, when combined with the existing extracellular matrix, initiates mild reactions in the tissues and induces the differentiation of bone formation induction cells [69,73]. The mild reaction in nearby tissue and the endocytosis process of carbon nanotube degradation form better osteointegration between normal bone and the critical-sized bone defect site. Moreover, MWNTs cause decreased osteoclastic activity and bone resorption from ectopic bone formation [74]. Its surface behavior also induces the differentiation of osteoblasts and causes mineralization [75,76]. Mineralization increases as the percentage of carbon nanotubes increases from 0.75 to 3 wt.% [72]. In this study, where 0.1% was used, better vasculogenesis was observed, though not in line with the previous report. The BMP-2 doped scaffold and the stem cell-alone-seeded scaffold showed bone tissue lamellar formation, with the BMP-2-alone group exhibiting much cartilaginous tissue. Perhaps the carbon nanotube increases the cartilaginous markers on human adipose tissue-derived mesenchymal stem cells, as reported by Valiani et al. (2014) [77]. The exogenous BMP-2 targets the periosteum and promotes chondrogenic lineage differentiation [78].

The scaffold loaded with both BMP-2 and stem cells demonstrated superior osteogenesis, characterized by a densely packed collagen matrix. Despite its constructive healing effects, evidence of the remodeling process was still observed, with no presence of inflammatory reactions. The combination of stem cells, BMP-2, and the nanocomposite biomimetic scaffold proved successful in achieving bone tissue regeneration in long bone critical-size defects, showing a faster and more organized manner of healing. The percentage of connective tissue was lower and mineralized matured regions were more pronounced, implying that the addition of stem cells and the bone growth factor BMP-2 enhances mature bone and collagen formation by the 90th day. On the 90th day, the stem cell and BMP-2 group exhibited better mineralization than other groups, with no adverse effects of the scaffold observed in vivo. This lack of adverse effects can be attributed to the absence of fibrosis, extensive osteoclasts, and inflammatory or infectious agents in the healed critical-size defect. Similar findings were observed in olecranon defects treated with a PCL-HAP and alginate composite. The use of multiwalled carbon nanotubes promotes mineralization and transitions from fibrous tissue union to mature bone formation in 3D bio-printed scaffolds [79]. In this experimental study, no scaffold remnants were found in any group at the 90th postoperative microphotometric analysis, likely due to the low percentage of biocompatible carbon nanotube and hydroxyapatite, causing the nanocomposite to absorb faster than other materials. The study reported that nanofibers making up the scaffold degrade and biodegrade faster [80].

The SEM analysis in the present study revealed distinctive features among the groups. In the control group, collagen fibrils were observed without mineralized bone formation at the defect site. The PCL + HAP and PCL + HAP + MWCNT-COOH groups exhibited irregularly arranged collagen fibrous tissue and minimal mineralized areas at the defect site. In the PCL + HAP group, certain areas of collagenous structures were relatively more intricate and well-connected with surrounding tissues. The PCL + HAP + MWCNT-COOH group showed the presence of a more abundant network of fibrous tissue along with an osseous callus, indicating the initial stages of calcification. The groups receiving stem cells and BMP2 along with the scaffolds demonstrated new bone formation, vascular tissue, and increased marrow space. The stem cell-loaded group exhibited higher compact tissue integration than the BMP2 alone group. Group F, receiving both stem cells and the growth factor, depicted high compactness, increased mineral area, and less marrow space than other groups. Some areas of group F showed pits, indicating sites of resorption and suggesting bone formation under remodeling processes, a finding supported by Singh et al. (2000) during the bone healing process [81].

The SEM study results were substantiated by the nanocomposite material’s physicochemical characteristics, with desirable porosity and interconnectivity enhancing interior bone tissue formation [82]. This is in line with longer biostability, improved scaffold kinetics, fast dissolution, and material fragmentation. The addition of stem cells and BMP2 supported bone formation in the critical-size defect, with the scaffold guiding neoformation and mineralization of bone tissue. Furthermore, scaffold characteristics supported vascularization and cellular regeneration [83]. Our findings align with Mohammed et al. (2019) [84], where a nanocomposite loaded with stem cells alone and a nanocomposite scaffold with BMP2 evidenced moderate bone formation compared to control and scaffold-alone groups. No scaffold remnants were observed in this study on the 90th day, possibly due to faster scaffold degradation in vivo. In contrast, the presence of hydroxyapatite particles was noticed on the 60th postoperative day of bone healing [81], attributed to the short study duration and higher scaffold material percentage. Ultimately, the SEM analysis supports the use of PCL + HAP + MWCNT-COOH along with stem cells and BMP2 for better early neoformation of bone tissue, and these SEM observations align with the radiographic and histopathological findings in this study.

## 5. Conclusions

In conclusion, our study evaluated various treatment approaches for critical-sized bone defects in rabbits. Group A, the control, demonstrated no signs of healing in the 15 mm radial diaphyseal defect after 90 days. However, the introduction of the PCL + HAP scaffold in Group B showed some improvement, and further enhancement was observed in Group C with the incorporation of the PCL + HAP + MWCNT-COOH scaffold. Notably, Group D, which received local injections of BMP-2, exhibited accelerated healing within 90 days. Combining rBMSC with PCL + HAP + MWCNT-COOH scaffold in Group E and administering additional BMP-2 in Group F resulted in an extensive acceleration of the osteogenic process. In summary, our findings suggest an ascending order of bone healing quality in critical-sized bone defects as follows: No Treatment < PCL + HAP Scaffold < PCL + HAP + MWCNT-COOH Scaffold < PCL + HAP + MWCNT-COOH Scaffold + BMP-2 < rBMSC-seeded PCL + HAP + MWCNT-COOH Scaffold < rBMSC-seeded PCL + HAP + MWCNT-COOH Scaffold + BMP-2.

Incorporating growth factor BMP-2 in a tissue-engineered rBMSC-loaded nanocomposite PCL + HAP + MWCNT-COOH construct can augment the osteoinductive and osteoconductive properties of the scaffold, thereby enhancing bone regeneration in critical-sized bone defects. By locally delivering BMP-2 to the defect site, the bone healing process was initiated and accelerated. Meanwhile, rBMSC had the ability to differentiate into various cell types, including osteoblasts, and also exerted paracrine effects by secreting growth factors that promoted tissue repair and regeneration. When seeded onto the nano scaffold, rBMSC created a conducive microenvironment for cell attachment, proliferation, and differentiation, enhancing their regenerative potential. Additionally, the nano scaffold provided structural support and mimicked the extracellular matrix, further facilitating tissue regeneration. Therefore, the combination of BMP-2 and rBMSC on a nano scaffold used in our study likely synergized to promote bone healing in critical-sized defects. This novel stem cell-loaded composite scaffold could prove worthy in the clinical management of nonunion and delayed union fractures in the near future.

## Figures and Tables

**Table 1:** In vivo experimental design of different groups included in this study.

Group	Animals	Treatment Protocol	Specimen Collection
A (Control)	6	External coaptation-splinting (ECS)	Day 90 PO
B	6	Polycaprolactone (PCL) + Hydroxyapatite (HAP) + ECS	Day 90 PO
C	6	PCL + HAP + MWCNT-COOH + ECS	Day 90 PO
D	6	PCL + HAP + MWCNT-COOH + Bone Morphogenetic Protein 2 (BMP-2) + ECS	Day 90 PO
E	6	PCL + HAP + MWCNT-COOH + rBMSC + ECS	Day 90 PO
F	6	PCL + HAP + MWCNT-COOH + rBMSC + BMP-2 + ECS	Day 90 PO

ECS: external coaptation-splinting; PCL: polycaprolactone; HAP: hydroxyapatite; MWCNT-COOH: carboxylated multiwalled carbon nanotube; BMP-2: bone morphogenetic protein 2; PO: postoperatively; rBMSC: rabbit bone marrow-derived mesenchymal stem cells.

## Data Availability

The original contributions presented in the study are included in the article/Supplementary Materials; further inquiries can be directed to the corresponding author.

## Supplementary Materials

The following supporting information can be downloaded at https://www.mdpi.com/article/10.3390/jfb15030066/s1. Table S1: Clinical observational parameters score. Table S2: Lameness scoring. Table S3: Modified radiographic score by (Yang et al., 1994). Table S4: Modified Lane and Sandhu (1987) and Heiple et al. (1987) histopathological scoring system. Table S5: The mean ± SD of heart rate of various treatment groups at different time intervals. Table S6: The mean ± SD of respiratory rate of various treatment groups at different time intervals. Table S7: The mean ± SD of rectal temperature of various treatment groups at different time intervals. Table S8: The mean ± SD of pain score of various treatment groups at different time intervals. Table S9: The mean ± SD of swelling score of various treatment groups at different time intervals. Table S10: The mean ± SD of exudation score of various treatment groups at different time intervals. Table S11: The mean ± SD of lameness score of various treatment groups at different time intervals. Table S12: The mean ± SD of left hind foot stride length (cm) of various treatment groups at different time intervals. Table S13: The mean ± SD of right hind foot stride length (cm) of various treatment groups at different time intervals. Table S14: The mean ± SD of left forefoot stride length (cm) of various treatment groups at different time intervals. Table S15: The mean ± SD of right fore foot stride length (cm) of various treatment groups at different time intervals. Table S16: The mean ± SD of fore foot step length (cm) of various treatment groups at different time intervals. Table S17: The mean ± SD of drag mark length (cm) of various treatment groups at different time intervals. Table S18: The mean ± SD of blood glucose (mg/dL) of various treatment groups at different time intervals. Table S19: The mean ± SD of serum total protein (g/dL) of various treatment groups at different time intervals. Table S20: The mean ± SD of serum alkaline phosphatase (IU/L) of various treatment groups at different time intervals. Table S21: The mean ± SD of serum phosphorus (mg/dL) of various treatment groups at different time intervals. Table S22: The mean ± SD of serum ionized calcium (mg/dL) of various treatment groups at different time intervals. Table S23: The mean ± SD of roentgenogram periosteal reaction score of various treatment groups at different time intervals. Table S24: The mean ± SD of osteotomy line score of various treatment groups at different time intervals. Table S25: The mean ± SD of critical size defect filling score of various treatment groups at different time intervals. Table S26: The mean ± SD of microphotometric analysis of 90th day in different treatment groups.
