# Supplementary material for: Bone Marrow-Derived Mesenchymal Stem Cell-Laden Nanocomposite Scaffolds Enhance Bone Regeneration in Rabbit Critical-Size Segmental Bone Defect Model
Source: J Funct Biomater. 2024 Mar 10;15(3):66. doi: 10.3390/jfb15030066 (PMC10971049; doi:10.3390/jfb15030066)
Supplement: Supplementary file 1 [file jfb-15-00066-s001.zip › jfb-2895201-supplementary.pdf]

Supplementary materials

Table S1: Clinical observational parameters score.

**Observational parameters**	**Score**			
	**0**	**1**	**2**	**3**
**Pain score**	None	Mild (occasional vocalization)	Moderate (frequent vocalization)	Severe (vociferous vocalization, withdraws limb, bites, struggles)
**Swelling score**	None	Mild	Obvious	N/A*
**Exudation score**	None	Mild (single 2×2 gauze wipe sufficient)	Moderate (two 2×2 gauze wipe sufficient)	Severe (three or more 2×2 gauze wipe used)

*N/A: Not Applicable

Table S2: Lameness scoring.

**Lameness score**	**Observations**
0	No lameness observed
1	Mild: Weight bearing lameness
2	Moderate: Weight bearing lameness with intermittent non-weight bearing
3	Severe: Non-weight bearing lameness with brief intermittent weight bearing
4	Non-weight bearing lameness at all times

Table S3: Modified radiographic score by (Yang et al., 1994).

Grading category	Score
**Periosteal reaction**	
None	0
Minimal (localized to the gaps)	1
Medium (extends over the gaps or towards ulna)	2
Moderate (1/4 to 1/2 of the defect area)	3
Full (1/2 to full length of defect)	4
**Osteotomy line**	
Both the osteotomy lines completely radiolucent	0
One of the osteotomy lines partially radiolucent	1
Both the osteotomy lines partially radiolucent	2
One of the osteotomy lines invisible	3
Both the osteotomy lines invisible	4
**Construct appearance (*not applicable)**	
Unchanged/intact	0
Mild resorption (one localized area)	1
Moderate resorption (more than one localized area	2
Mostly replaced	3
Fully replaced	4
**Defect filling score**	
No change in critical sized defect	0
1-25% CSD filled with bony/cartilaginous tissue	1
25-50% CSD filled with bony/cartilaginous tissue	2
50-75% CSD filled with bony/cartilaginous tissue	3
75-100% CSD	4

*The experiment scaffold was radiolucent in skiagram. Hence evaluation of construct appearance was not applicable.

Table S4: Modified Lane and Sandhu (1987) and Heiple et al. (1987) histopathological scoring system.

Sl. No	Feature	Score
**Histopathological scoring system**		
**A**	**Osteogenesis**	
1	No osteogenesis	0
2	Weak osteogenesis	1
3	Medium osteogenesis	2
4	Good osteogenesis	3
5	Perfect osteogenesis	4
**B**	**Union**	
1	No evidence of union	0
2	Fibrous union	1
3	Osteochondral union	2
4	Bony union	3
5	Complete organization of graft	4
**C**	**Marrow**	
1	None in the resected area	0
2	Beginning to appear	1
3	Present in greater than 1 /2 defect	2
4	Complete colonization by red marrow	3
5	Mature fatty marrow	4
**D**	**Cancellous bone/medullary bone**	
1	No osseous cellular activity	0
2	Early apposition of new bone	1
3	Active apposition of new bone	2
4	Reorganizing cancellous bone	3
5	Complete re-organization of cancellous bone	4
**E**	**Cortical/compact bone**	
1	None	0
2	Early appearance	1
3	Formation underway	2
4	Mostly re-organized	3
5	Completely formed	4

Table S5: The mean±SD of heart rate of various treatment groups at different time intervals.

Group	Time interval in days							
	Pre	Day 1	Day 2	Day 3	Day 4	Day 5	Day 6	Day 7
A	247.5±14.82^{a}	250±48.76^{a}	246.83±10.36^{a}	250±22.52^{a}	258±25.95^{a}	229.67±21.74^{a}	245±20.93^{a}	246.5±23.71^{a}
B	248.83±28.27^{a}	258.5±20.7^{a}	249.17±13.79^{a}	247.67±46.37^{a}	243.33±9.97^{a}	256.67±27.62^{a}	256.83±21.14^{a}	235.83±34.16^{a}
C	248.83±21.89	259.17±24.55^{a}	258.33±21.63^{a}	240.83±24.24^{a}	268.67±24.8^{a}	247±24.66^{a}	236.67±30.71^{a}	245.83±21.4^{a}
D	248.67±36.06^{a}	244.5±23.26^{a}	260.67±27.22^{a}	247±20.75^{a}	248.33±36.81^{a}	251±22.2^{a}	250.67±34.62^{a}	243.67±23.28^{a}
E	240.67±16.44^{a}	241.17±16.47^{a}	236.5±16.57^{a}	231.83±21.66^{a}	223.67±35.83^{a}	245.83±20.02^{a}	252±24.27^{a}	249.33±21.37^{a}
F	252.83±25.66^{a}	248.83±21.79^{a}	233±23.17^{a}	234.83±41.94^{a}	253.33±19.91^{a}	247.17±21.08^{a}	226.17±22.97^{a}	247.83±22.64^{a}

The different alphabet and * in superscripts represents significant between groups and within group comparison at different time intervals respectively. Different alphabets means $p < 0.05$, "*" means $p < 0.05$, "**" means $p < 0.01$.

Table S6: The mean±SD of respiratory rate of various treatment groups at different time intervals.

Group	Time interval in days							
	Pre	Day 1	Day 2	Day 3	Day 4	Day 5	Day 6	Day 7
A	50.83±7.6^{a}	53.33±6.86^{a}	48.5±13.14^{a}	48±11.4^{a}	46.67±11.33^{a}	48.5±11.84^{a}	48.67±7.47^{a}	55.33±10.56^{a}
B	58±10.35^{a}	49.5±11.15^{a}	50±9.38^{a}	53.17±7.63^{a}	53.17±9.91^{a}	43.67±6.89^{a}	47.17±13.45^{a}	48.5±5.96
C	49.83±9.68^{a}	51.5±11.84^{a}	52.33±7.58^{a}	49.5±9.57^{a}	47.33±8.12^{a}	45.5±9.54^{a}	47±10.14	46±6.99^{a}
D	50.17±8.42^{a}	53±8.34^{a}	47.5±15.42^{a}	52.17±7.96^{a}	51.17±9.2^{a}	46±10.24^{a}	53.67±7.47^{a}	49.5±10.33^{a}
E	55±9.36^{a}	55.17±10.4^{a}	53.17±12.3^{a}	50.33±9.18^{a}	52.5±7.01^{a}	51.5±11.43^{a}	55.33±7.63^{a}	49.83±6.55^{a}
F	47.33±8.04^{a}	51±6.69^{a}	54.67±10.82^{a}	43±10.75^{a}	51±9.34^{a}	53.67±13.6^{a}	45.67±11.99^{a}	44.5±10.37^{a}

The different alphabet and * in superscripts represents significant between groups and within group comparison at different time intervals respectively. Different alphabets means $p < 0.05$, "*" means $p < 0.05$, "**" means $p < 0.01$.

Table S7: The mean±SD of rectal temperature of various treatment groups at different time intervals.

Group	Time interval in days							
	Pre	Day 1	Day 2	Day 3	Day 4	Day 5	Day 6	Day 7
A	101.77±0.66^{a}	101.48±0.5^{a}	101.82±0.62^{a}	101.85±0.58^{a}	102±0.76^{a}	101.93±0.84^{a}	86.58±37.39^{a}	101.7±0.75^{a}
B	101.18±0.9^{a}	101.63±0.39^{a}	101.28±0.84^{a}	101.35±0.82^{a}	101.22±0.79^{a}	101.22±0.79^{a}	101.33±0.65^{a}	100.98±0.78^{a}
C	101.5±0.76^{a}	101.07±0.31^{a}	101.65±0.72^{a}	101.68±0.83^{a}	101.62±0.78^{a}	101.62±0.78^{a}	101.57±0.74^{a}	101.68±0.85^{a}
D	100.92±1.01^{a}	101.8±0.85^{a}	101.47±0.77^{a}	101.62±0.73^{a}	101.25±0.71^{a}	101.25±0.71^{a}	101.27±0.44^{a}	101.52±0.61^{a}
E	101.38±1.29^{a}	100.87±1.01^{a}	101.58±0.75^{a}	101.43±0.94^{a}	101.53±0.98^{a}	101.53±0.98^{a}	101.45±0.98^{a}	101.47±0.82^{a}
F	101.32±0.64^{a}	101.07±0.85^{a}	101.8±0.54^{a}	101.52±0.6^{a}	101.47±0.61^{a}	101.47±0.61^{a}	101.53±0.67^{a}	101.6±0.49^{a}

The different alphabet and * in superscripts represents significant between groups and within group comparison at different time intervals respectively. Different alphabets means $p < 0.05$, "*" means $p < 0.05$, "**" means $p < 0.01$.

Table S8: The mean±SD of pain score of various treatment groups at different time intervals.

Group	Time interval in days												
	Pre	Day 1	Day 2	Day 3	Day 4	Day 5	Day 6	Day 7	Day 8	Day 9	Day 10	Day 30	Day 60	Day 90
A	0±0	3±0*	3±0*	3±0*	3±0*	3±0*	2.83±0.41^{a*}	2.5±0.55*	2.5±0.55*	2.33±0.52*	2.33±0.52^{a*}	0.83±0.41*	0.5±0.55^{a}	0.33±0.52
B	0±0	3±0*	3±0*	3±0*	2.83±0.41*	3±0*	2±0^{b*}	2±0*	2±0*	2±0*	2.17±0.41^{b*}	0±0	0±0^{b}	0±0
C	0±0	3±0*	3±0*	3±0*	2.83±0.41*	3±0*	2.17±0.41^{b*}	2.17±0.41*	2.17±0.41*	2.17±0.41*	2.17±0.41^{b*}	0±0	0±0^{ab}	0±0
D	0±0	3±0*	3±0*	3±0*	2.83±0.41*	3±0*	2.17±0.41^{b*}	2.33±0.52*	2±0*	2.33±0.52*	2.33±0.52^{b*}	0±0	0.17±0.41^{ab}	0±0
E	0±0	3±0*	3±0*	3±0*	2.83±0.41*	2.83±0.41*	2±0^{b*}	2.17±0.41*	2±0*	2±0*	2.33±0.52^{ab*}	0.33±0.52	0.33±0.52^{ab}	0±0
F	0±0	3±0*	3±0*	3±0*	2.83±0.41*	3±0*	2±0^{b*}	2.17±0.41*	2±0*	2.17±0.41*	2.17±0.41^{ab*}	0.33±0.52	0.5±0.55^{ab}	0±0

The different alphabet and * in superscripts represents significant between groups and within group comparison at different time intervals respectively. Different alphabets means $p< 0.05$, "*" means $p< 0.05$, "**" means $p< 0.01$.

Table S9: The mean±SD of swelling score of various treatment groups at different time intervals.

Group	Time interval in days												
	Pre	Day 1	Day 2	Day 3	Day 4	Day 5	Day 6	Day 7	Day 8	Day 9	Day 10	Day 30	Day 60	Day 90
A	0±0	1.67±0.52*	1.5±0.55*	1.33±0.52*	1.33±0.52*	1±0*	0.5±0.55	0.33±0.52	0.33±0.52	0.17±0.41	0±0	0±0	0±0	0±0
B	0±0	1.17±0.41*	1.33±0.52*	1.17±0.41*	1.33±0.52*	0.83±0.41*	0.33±0.52	0.17±0.41	0±0	0±0	0±0	0±0	0±0	0±0
C	0±0	1.33±0.52*	1.17±0.41*	1±0*	1±0*	1±0*	0±0	0±0	0±0	0±0	0±0	0±0	0±0	0±0
D	0±0	1.33±0.52*	1.17±0.41*	1.33±0.52*	1.33±0.52*	1.17±0.41*	0±0	0.17±0.41	0±0	0±0	0±0	0±0	0±0	0±0
E	0±0	1.33±0.52*	1.67±0.52*	1±0*	1±0*	1±0*	0±0	0±0	0±0	0±0	0±0	0±0	0±0	0±0
F	0±0	1.33±0.52*	1.33±0.52*	1±0*	1±0*	1±0*	0±0	0±0	0±0	0±0	0±0	0±0	0±0	0±0

The different alphabet and * in superscripts represents significant between groups and within group comparison at different time intervals respectively. Different alphabets means $p< 0.05$, "*" means $p< 0.05$, "**" means $p< 0.01$.

Table S10: The mean±SD of exudation score of various treatment groups at different time intervals.

Group	Time interval in days												
	Pre	Day 1	Day 2	Day 3	Day 4	Day 5	Day 6	Day 7	Day 8	Day 9	Day 10	Day 30	Day 60	Day 90
A	0±0	2.17±0.75*	2.5±0.55^{ab*}	2.67±0.52^{a*}	2.17±0.41^{a*}	1.17±0.41*	0.83±0.41^{a*}	0.5±0.55	0.33±0.52	0.17±0.41	0±0	0±0	0±0	0±0
B	0±0	2±0.89*	2.17±0.41^{a*}	1.83±0.98^{ab*}	1.67±0.82^{ab*}	1.33±0.52*	0.67±0.52^{ab*}	0.17±0.41	0±0	0±0	0±0	0±0	0±0	0±0
C	0±0	1.83±0.75*	2.33±0.52^{a*}	1.83±0.98^{ab*}	1.17±0.41^{b*}	1.17±0.41*	0.67±0.52^{ab*}	0.17±0.41	0±0	0±0	0±0	0±0	0±0	0±0
D	0±0	1.67±0.82*	1.17±0.75^{b*}	1.17±0.75^{b*}	0.83±0.75^{b}	0.83±0.75	0.17±0.41^{b}	0.17±0.41	0±0	0±0	0±0	0±0	0±0	0±0
E	0±0	1.83±0.75*	1.67±0.82^{ab*}	1.5±0.55^{b*}	1.17±0.41^{b*}	1.17±0.41*	0.17±0.41^{b}	0.17±0.41	0.17±0.41	0.17±0.41	0.17±0.41	0±0	0±0	0±0
F	0±0	1.83±0.75*	1.5±0.84^{ab*}	1.33±0.52^{b*}	1±0.63^{b*}	1±0.63*	0.33±0.52^{ab}	0.33±0.52	0.17±0.41	0±0	0±0	0±0	0±0	0±0

The different alphabet and * in superscripts represents significant between groups and within group comparison at different time intervals respectively. Different alphabets means $p< 0.05$, "*" means $p< 0.05$, "**" means $p< 0.01$.

Table S11: The mean±SD of lameness score of various treatment groups at different time intervals.

Group	Time interval in days						
	Pre	Day 1	Day 15	Day 30	Day 45	Day 60	Day 90
A	0±0	4±0*	4±0*	4±0*	3.83±0.41^{a*}	3.5±0.55^{a*}	2.5±0.55^{a*}
B	0±0	4±0*	3.67±0.52*	3.67±0.52*	3.5±0.55^{ab*}	2.83±0.41^{b*}	2±0.89^{a*}
C	0±0	4±0*	3.83±0.41*	3.83±0.41*	3.33±0.52^{ab*}	2.5±0.55^{b*}	1.83±0.98^{a*}
D	0±0	4±0*	3.83±0.41*	3.67±0.52*	3.33±0.52^{ab*}	2.17±0.75^{bc*}	1.33±0.82^{ab*}
E	0±0	4±0*	3.83±0.41*	3.67±0.52*	3.5±0.55^{ab*}	2.33±0.52^{b*}	1±0.89^{ab}
F	0±0	4±0*	3.83±0.41*	3.67±0.52*	3±0.63^{b*}	1.5±0.55^{c*}	0.5±0.55^{b}

The different alphabet and * in superscripts represents significant between groups and within group comparison at different time intervals respectively. Different alphabets means $p < 0.05$, "*" means $p < 0.05$, "**" means $p < 0.01$.

Table S12: The mean±SD of left hind foot stride length (cm) of various treatment groups at different time intervals.

Group	Time interval in days						
	Pre	Day 1	Day 15	Day 30	Day 45	Day 60	Day 90
A	31.83±1.17^{a}	31.42±0.92^{a}	30.33±1.21^{a}	30.25±1.08^{a}	30.25±1.25^{a}	28.83±6.05^{a}	31.25±0.88^{a}
B	31.33±1.37^{a}	29.97±1.33^{a}	28.42±5.92^{a}	30.83±1.21^{a}	29.58±1.56^{a}	30.25±2.19^{a}	31.67±3.39^{a}
C	32.17±0.52^{a}	30.17±1.91^{a}	31.5±1.26^{a}	31.25±0.99^{a}	31±1.41^{a}	31.75±3.34^{a}	31.67±0.41^{a}
D	30.83±1.81^{a}	29.42±2.2^{a}	28.83±0.75^{a}	28.92±1.2^{a}	29.25±2.36^{a}	28.25±1.08^{a}	28.83±2.04^{a}
E	30.92±1.86^{a}	30.08±1.16^{a}	29.33±1.99^{a}	30.08±1.16^{a}	29.83±1.17^{a}	29.92±0.92^{a}	30.42±1.11^{a}
F	32±0.71^{a}	30.17±0.82^{a}	29.75±0.69^{a}	29.5±1.9^{a}	30.25±1.08^{a}	30.17±0.75^{a}	30.17±0.98^{a}

The different alphabet and * in superscripts represents significant between groups and within group comparison at different time intervals respectively. Different alphabets means $p < 0.05$, "*" means $p < 0.05$, "**" means $p < 0.01$.

Table S13: The mean±SD of right hind foot stride length (cm) of various treatment groups at different time intervals.

Group	Time interval in days						
	Pre	Day 1	Day 15	Day 30	Day 45	Day 60	Day 90
A	31.83±1.17^{a}	30.42±0.8^{a}	30±1.26^{a}	30.58±0.92^{a}	30.25±1.25^{a}	27.5±6.72^{a}	30.58±0.92^{a}
B	30.83±1.33^{a}	29.97±1.33^{a}	27.75±5.77a	29.83±1.13^{a}	29.83±1.72^{a}	30±2^{a}	30.08±0.92^{a}
C	30.83±1.44^{a}	29.42±1.28^{a}	29.92±1.36^{a}	29.33±1.03^{a}	29.58±1.11^{a}	31.92±3.41^{a}	29.5±1.05^{a}
D	29.08+2.65^{a}	28.33+1.54^{a}	28.75+1.6^{a}	28.42+1.5^{a}	29.83±1.72^{a}	28.58+1.28^{a}	28.5+1.52^{a}
E	30±1.48^{a}	29.17±2.07^{a}	28.58±2.11^{a}	28.83±1.63^{a}	29.42±1.63^{a}	29±1.41^{a}	29±1.52^{a}
F	31.25±1.33^{a}	30.17±1.21^{a}	30.08±1.16^{a}	30.17±1.21^{a}	30±1.26^{a}	30.08±1.02^{a}	30.67±1.03^{a}

The different alphabet and * in superscripts represents significant between groups and within group comparison at different time intervals respectively. Different alphabets means $p < 0.05$, "*" means $p < 0.05$, "**" means $p < 0.01$.

Table S14: The mean±SD of left forefoot stride length (cm) of various treatment groups at different time intervals.

Group	Time interval in days						
	Pre	Day 1	Day 15	Day 30	Day 45	Day 60	Day 90
A	30.67 ± 1.21^{a}	30.42 ± 0.8^{a}	30.5 ± 0.84^{a}	30.5 ± 0.84^{a}	30.33 ± 1.03^{a}	30.33 ± 1.03^{a}	30.58 ± 0.92^{a}
B	30.83 ± 1.33^{a}	29.75 ± 1.17^{a}	29.83 ± 1.13^{a}	29.83 ± 1.13^{a}	29.83 ± 1.17^{a}	29.83 ± 0.98^{a}	30.08 ± 0.92^{a}
C	29.83 ± 0.93^{a}	29.42 ± 1.28^{a}	29.5 ± 1.22^{a}	29.5 ± 1.22^{a}	29.58 ± 1.11^{a}	29.5 ± 1.05^{a}	29.5 ± 1.05^{a}
D	28.83 ± 1.72^{a}	28.33 ± 1.54^{a}	28.5 ± 1.55^{a}	28.33 ± 1.54^{a}	28.33 ± 1.37^{a}	28.58 ± 1.28^{a}	28.75 ± 1.54^{a}
E	29.08 ± 1.88^{a}	28.33 ± 1.66^{a}	28.67 ± 1.25^{a}	28.33 ± 1.66^{a}	28.33 ± 1.4^{a}	28.42 ± 1.11^{a}	28.83 ± 1.63^{a}
F	29.5 ± 0.89^{a}	29.67 ± 0.88^{a}	29.58 ± 0.8^{a}	29.67 ± 0.88^{a}	29.58 ± 0.8^{a}	29.67 ± 0.82^{a}	30.17 ± 0.98^{a}

The different alphabet and * in superscripts represents significant between groups and within group comparison at different time intervals respectively. Different alphabets means $p < 0.05$, "*" means $p < 0.05$, "**" means $p < 0.01$.

Table S15: The mean±SD of right fore foot stride length (cm) of various treatment groups at different time intervals.

Group	Time interval in days						
	Pre	Day 1	Day 15	Day 30	Day 45	Day 60	Day 90
A	30.17 ± 3.31^{a}	30.17 ± 3.5	$25.92\pm3.31^{*}$	$22\pm2.68^{**}$	$20.25\pm5.96^{**}$	$22.83\pm3.37^{a**}$	$24\pm2.97^{a**}$
B	30 ± 1.26^{a}	29.25 ± 2.72	$25.67\pm4.23^{*}$	$25.75\pm2.27^{*}$	$23.58\pm1.5^{*}$	$23.92\pm3.58^{ab*}$	$26.92\pm1.96^{ab**}$
C	30.17 ± 1.57^{a}	$33.75\pm2.82^{**}$	27.67 ± 2.4	$25.42\pm1.11^{*}$	$24.08\pm2.25^{**}$	27.58 ± 2.54^{bc}	29.33 ± 1.03^{b}
D	27.83 ± 1.47^{a}	30.25 ± 2.4	24.17 ± 2.8	24.08 ± 2.97	23 ± 1.92	31.25 ± 1.54^{c}	28.5 ± 1.52^{b}
E	28.33 ± 1.37^{a}	$31.25\pm2.32^{*}$	$23.92\pm2.71^{*}$	$23.75\pm1.97^{*}$	23.5 ± 2.05	24.83 ± 1.29^{ab}	28.33 ± 0.98^{b}
F	29.5 ± 0.89^{a}	32 ± 2.3	$24\pm2.47^{**}$	$23.92\pm1.91^{**}$	$23.17\pm1.51^{**}$	26.17 ± 1.94^{ab}	30.17 ± 0.98^{b}

The different alphabet and * in superscripts represents significant between groups and within group comparison at different time intervals respectively. Different alphabets means $p < 0.05$, "*" means $p < 0.05$, "**" means $p < 0.01$.

Table S16: The mean±SD of fore foot step length (cm) of various treatment groups at different time intervals.

Group	Time interval in days						
	Pre	Day 1	Day 15	Day 30	Day 45	Day 60	Day 90
A	9 ± 1.41^{a}	$4.86\pm1.75^{a**}$	$4.29\pm1.11^{a**}$	$4.43\pm1.17^{a**}$	$4.21\pm1.52^{a**}$	$5.21\pm1.63^{a**}$	$6\pm1.12^{a**}$
B	8.42 ± 0.92^{a}	6.79 ± 1.31^{ab}	6.83 ± 1.63^{b}	7.67 ± 1.94^{b}	7.67 ± 1.89^{b}	7.92 ± 1.66^{b}	7.08 ± 1.36^{ab}
C	9.75 ± 1.08^{a}	$7.17\pm0.88^{b**}$	$7.29\pm1.42^{b**}$	$7.33\pm0.93^{b*}$	$7.42\pm1.8^{b*}$	8.67 ± 1.78^{b}	8.42 ± 0.8^{bc}
D	8.25 ± 1.13^{a}	7.08 ± 0.97^{b}	7.08 ± 0.97^{b}	7.33 ± 1.4^{b}	7.42 ± 0.58^{b}	7.5 ± 1.38^{ab}	8.17 ± 1.29^{bc}
E	9.08 ± 1.53^{a}	7.83 ± 1.13^{b}	8.17 ± 0.93^{b}	7.5 ± 1.05^{b}	8.83 ± 1.57^{b}	8.75 ± 1.21^{b}	9.42 ± 1.11^{c}
F	9.08 ± 0.66^{a}	7.92 ± 0.86^{b}	8.08 ± 0.38^{b}	7.92 ± 0.58^{b}	7.75 ± 0.42^{b}	8 ± 0.71^{b}	8.25 ± 0.82^{bc}

The different alphabet and * in superscripts represents significant between groups and within group comparison at different time intervals respectively. Different alphabets means $p < 0.05$, "*" means $p < 0.05$, "**" means $p < 0.01$.

Table S17: The mean±SD of drag mark length (cm) of various treatment groups at different time intervals.

Group	Time interval in days						
	Pre	Day 1	Day 15	Day 30	Day 45	Day 60	Day 90
A	$0±0^{a}$	$22.92±2.85^{a**}$	$3.5±1.38^{a**}$	$3±0.95^{a**}$	$2.25±1.86^{a**}$	$1.33±1.54^{a**}$	$0.33±0.82^{a}$
B	$0±0^{a}$	$9.17±7.93^{b*}$	$0±0^{b}$	$0±0^{b}$	$0±0^{b}$	$0±0^{b}$	$0±0^{a}$
C	$0±0^{a}$	$5.92±7^{b}$	$1.58±3.88^{b}$	$0±0^{b}$	$0±0^{b}$	$0±0^{b}$	$0±0^{a}$
D	$0±0^{a}$	$7.75±7.03^{b}$	$0±0^{b}$	$0±0^{b}$	$0±0^{b}$	$0±0^{b}$	$0±0^{a}$
E	$0±0^{a}$	$4.67±5.27_{b}$	$0±0^{b}$	$0±0^{b}$	$0±0^{b}$	$0±0^{b}$	$0±0^{a}$
F	$0±0^{a}$	$4.5±5.23^{b}$	$0±0^{b}$	$0±0^{b}$	$0±0^{b}$	$0±0^{b}$	$0±0^{a}$

The different alphabet and * in superscripts represents significant between groups and within group comparison at different time intervals respectively. Different alphabets means $p < 0.05$, "*" means $p < 0.05$, "**" means $p < 0.01$.

Table S18: The mean±SD of blood glucose (mg/dL) of various treatment groups at different time intervals.

Group	Day 0	Day 30	Day 60	Day 90
A	105.5±7.18	106±7.24	106±4.69	107.67±5.68
B	106.83±7.41	107.5±5.82	105.5±2.26	105.67±3.39
C	107.33±6.28	106.33±3.08	106.33±4.63	104.67±2.42
D	106±6.23	106±4.69	109.5±6.02	106.17±5.42
E	106.67±6.47	106.83±3.31	104±1.41	105.17±2.64
F	108.83±6.97	105±2.83	105.67±4.5	105.33±6.44

The different alphabet and * in superscripts represents significant between groups and within group comparison at different time intervals respectively. Different alphabets means $p < 0.05$, "*" means $p < 0.05$, "**" means $p < 0.01$.

Table S19: The mean±SD of serum total protein (g/dL) of various treatment groups at different time intervals.

Group	Day 0	Day 30	Day 60	Day 90
A	6.95±0.88	6.38±1.44	$5.95±0.68^{*}$	$5.64±0.69^{*}$
B	6.91±0.88	6.44±0.74	5.97±0.32	6.01±0.63
C	6.76±1.05	6.85±1.04	6.22±0.49	6.37±0.7
D	6.12±0.71	7.35±1.34	6.22±0.78	6.07±0.97
E	6.71±0.75	6.36±0.85	5.83±0.82	6.16±0.71
F	6.36±0.63	6.82±0.58	6.21±0.74	6.48±0.72

The different alphabet and * in superscripts represents significant between groups and within group comparison at different time intervals respectively. Different alphabets means $p < 0.05$, "*" means $p < 0.05$, "**" means $p < 0.01$.

Table S20: The mean±SD of serum alkaline phosphatase (IU/L) of various treatment groups at different time intervals.

Group	Day 0	Day 30	Day 60	Day 90
A	20.35±3.42^{a}	27.43±3.76^{a**}	34.24±5.24^{a**}	31±3.16^{a**}
B	19.99±2.36^{a}	25.58±4.77^{a*}	34.56±4.02^{a**}	32.56±3.35^{a**}
C	20.19±3.99^{a}	30±4.58^{ab**}	37.28±9.05^{a**}	30.82±7.45^{a**}
D	19.78±4.51^{a}	35.17±3.97^{b**}	59.73±5.28^{b**}	53.29±7.45^{b**}
E	18.16±2.33^{a}	28.47±4.75^{a*}	38.35±3.94^{a**}	37.86±7.06^{a**}
F	19.58±2.88^{a}	35.44±3.61^{b**}	61.58±4.67^{b**}	50.81±4.61^{b**}

The different alphabet and * in superscripts represents significant between groups and within group comparison at different time intervals respectively. Different alphabets means $p < 0.05$, "*" means $p < 0.05$, "**" means $p < 0.01$.

Table S21: The mean±SD of serum phosphorus (mg/dL) of various treatment groups at different time intervals.

Group	Day 0	Day 30	Day 60	Day 90
A	4.79±0.36^{a}	5.2±0.08^{a*}	5.11±0.14^{a}	4.74±0.16^{a}
B	4.68±0.38^{a}	5.53±0.14^{b**}	5.36±0.14^{a**}	5.16±0.23^{b}
C	4.56±0.29^{a}	5.36±0.06^{a**}	5.42±0.28^{a**}	5.2±0.18^{b**}
D	4.57±0.37^{a}	5.72±0.09^{cd**}	6±0.09^{bc**}	5.67±0.14^{c**}
E	4.65±0.36^{a}	5.62±0.11^{bc**}	5.84±0.15^{b**}	5.6±0.18^{c**}
F	4.77±0.28^{a}	5.89±0.10^{d**}	6.16±0.16^{c**}	5.88±0.11^{c**}

The different alphabet and * in superscripts represents significant between groups and within group comparison at different time intervals respectively. Different alphabets means $p < 0.05$, "*" means $p < 0.05$, "**" means $p < 0.01$.

Table S22: The mean±SD of serum ionized calcium (mg/dL) of various treatment groups at different time intervals.

Group	Day 0	Day 30	Day 60	Day 90
A	7.85±1.07^{a}	6.67±0.75^{a*}	5.28±1.1^{a**}	4.86±0.75^{a**}
B	6.81±1.78^{a}	5.96±0.85^{ac}	5.05±0.6^{a*}	4.6±0.7^{ab**}
C	7.27±1.36^{a}	5.4±1.23^{abc**}	5.42±0.66^{a**}	4.28±0.58^{ab**}
D	7.01±1.16^{a}	4.06±0.71^{bc**}	2.75±1.04^{b**}	3.39±1.35^{bc**}
E	7.53±0.62^{a}	4.82±0.78^{c**}	2.78±0.7^{b**}	3.18±0.7^{bc**}
F	7.13±1.34^{a}	4.03±0.72^{c**}	1.7±0.67^{b**}	1.96±0.38^{c**}

The different alphabet and * in superscripts represents significant between groups and within group comparison at different time intervals respectively. Different alphabets means $p < 0.05$, "*" means $p < 0.05$, "**" means $p < 0.01$.

Table S23: The mean±SD of roentgenogram periosteal reaction score of various treatment groups at different time intervals.

Group	Day 0	Day 30	Day 60	Day 90
A	0 ± 0^{a}	$0.33\pm0.52^{a*}$	$0.67\pm0.52^{a*}$	$0.83\pm0.41^{a*}$
B	0 ± 0^{a}	$0.67\pm0.52^{ab*}$	$1.5\pm0.55^{b*}$	$2\pm0^{b*}$
C	0 ± 0^{a}	$1\pm0^{b*}$	$1.83\pm0.41^{b*}$	$2.83\pm0.41^{c*}$
D	0 ± 0^{a}	$3\pm0^{ce*}$	$3.83\pm0.41^{ce*}$	$4\pm0^{df*}$
E	0 ± 0^{a}	$3\pm0^{de*}$	$4\pm0^{de*}$	$4\pm0^{ef*}$
F	0 ± 0^{a}	$3\pm0^{e*}$	$4\pm0^{e*}$	$4\pm0^{f*}$

The different alphabet and * in superscripts represents significant between groups and within group comparison at different time intervals respectively. Different alphabets means $p< 0.05$, "*" means $p< 0.05$, "**" means $p< 0.01$.

Table S24: The mean±SD of osteotomy line score of various treatment groups at different time intervals.

Group	Day 0	Day 30	Day 60	Day 90
A	0 ± 0^{a}	$0.67\pm0.52^{a*}$	$2\pm0^{a*}$	$2.83\pm0.41^{a*}$
B	0 ± 0^{a}	$1\pm0^{a*}$	$2.67\pm0.52^{b*}$	$2.83\pm0.41^{a*}$
C	0 ± 0^{a}	$2.67\pm0.52^{bc*}$	$4\pm0^{c*}$	$4\pm0^{b*}$
D	0 ± 0^{a}	$2.83\pm0.41^{c*}$	$4\pm0^{c*}$	$4\pm0^{b*}$
E	0 ± 0^{a}	$3.5\pm0.55^{d*}$	$4\pm0^{c*}$	$4\pm0^{b*}$
F	0 ± 0^{a}	$3.67\pm0.52^{d*}$	$4\pm0^{c*}$	$4\pm0^{b*}$

The different alphabet and * in superscripts represents significant between groups and within group comparison at different time intervals respectively. Different alphabets means $p< 0.05$, "*" means $p< 0.05$, "**" means $p< 0.01$.

Table S25: The mean±SD of critical size defect filling score of various treatment groups at different time intervals.

Group	Day 0	Day 30	Day 60	Day 90
A	0 ± 0^{a}	0 ± 0^{a}	0 ± 0^{a}	0 ± 0^{a}
B	0 ± 0^{a}	$1\pm0^{b*}$	$1\pm0^{b*}$	$1\pm0^{b*}$
C	0 ± 0^{a}	$1\pm0^{b*}$	$1\pm0^{b*}$	$1.83\pm0.41^{c*}$
D	0 ± 0^{a}	$1.17\pm0.41^{b*}$	$3\pm0^{c*}$	$3\pm0^{d*}$
E	0 ± 0^{a}	$2\pm0^{c*}$	$2.83\pm0.41^{c*}$	$4\pm0^{e*}$
F	0 ± 0^{a}	$2.33\pm0.52^{c*}$	$3.83\pm0.41^{d*}$	$4\pm0^{e*}$

The different alphabet and * in superscripts represents significant between groups and within group comparison at different time intervals respectively. Different alphabets means $p< 0.05$, "*" means $p< 0.05$, "**" means $p< 0.01$.

Table S26: The mean±SD of microphotometric analysis of 90th day in different treatment groups.

Group	Osteogenesis	Union	Marrow	Cancellous bone	Cortical bone	Total HP score
A	0±0[a]	0.17±0.41[a]	0±0[a]	0±0[a]	0±0[e]	0.17±0.41[a]
B	0.83±0.41[b]	1±0[b]	0±0[a]	0.83±0.41[b]	0±a[a]	2.67±0.82[b]
C	1.67±0.52[c]	1.17±0.41[b]	0±0[a]	1.67±0.52[c]	0.33±0.52[ab]	4.83±0.75[c]
D	2±0[c]	2.17±0.41[c]	0.33±0.52[ab]	2.83±0.41[d]	0.83±0.41[b]	8.17±0.75[d]
E	3.17±0.41[d]	3.33±0.52[d]	1.17±0.75[b]	3.83±0.41[e]	2.33±0.52[c]	13.83±1.47[e]
F	3.33±0.52[d]	3.5±0.55[d]	2±0.89[b]	3.83±0.41[e]	3.33±0.52[d]	16±1.1[f]

The different alphabet and * in superscripts represents significant between groups and within group comparison at different time intervals respectively. Different alphabets means $p < 0.05$, "*" means $p < 0.05$, "**" means $p < 0.01$.
